# Ratiometric pH-Responsive ^19^F Magnetic Resonance Imaging Contrast Agents Based on Hydrazone Switches

**DOI:** 10.1021/acs.analchem.1c04978

**Published:** 2022-02-14

**Authors:** Dawid Janasik, Krzysztof Jasiński, Władysław P. Węglarz, Ivan Nemec, Pawel Jewula, Tomasz Krawczyk

**Affiliations:** † Department of Chemical Organic Technology and Petrochemistry, Silesian University of Technology Krzywoustego 4,44-100 Gliwice, Poland; ‡ Institute of Nuclear Physics Polish Academy of Sciences, 31-342 Krakow, Poland; § Central European Institute of Technology Brno University of Technology, Purkyňova 123, 612-00 Brno, Czech Republic; ∥ Department of Inorganic Chemistry, Faculty of Science, Palacký University 17. Listopadu 1192/12, 771 46 Olomouc, Czech Republic

## Abstract

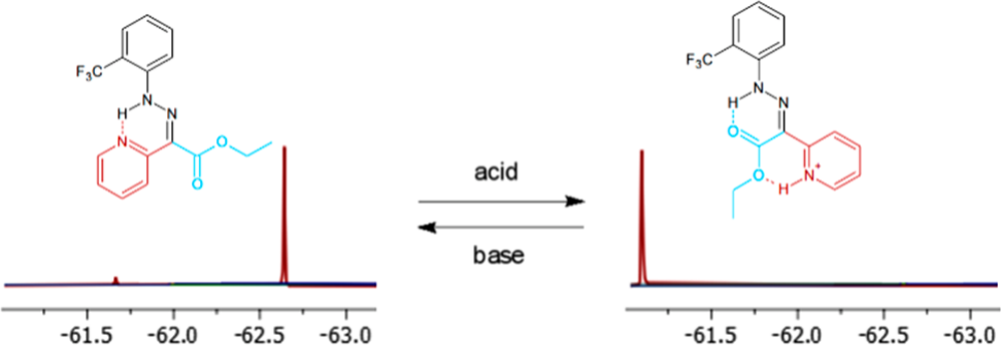

Hydrazone-based molecular switches serve as efficient ratiometric pH-sensitive agents that can be tracked with ^19^F NMR/MRI and ^1^H NMR. Structural changes induced between pH 3 and 4 lead to signal appearance and disappearance at ^1^H and ^19^F NMR spectra allowing ratiometric pH measurements. The most pronounced are resonances of the CF_3_ group shifted by 1.8 ppm with ^19^F NMR and a hydrazone proton shifted by 2 ppm with ^1^H NMR.

The measurement of pH is a fundamental aspect of chemical research. Many tools have been developed for this purpose, and visual indicators^1^ and pH-sensitive electrodes^2^ are commonplace in almost every laboratory. More sophisticated techniques were proposed for medicinal diagnostic, where a deviation from physiological pH indicates various pathological changes. For example, gastroesophageal reflux disease manifests an esophageal pH < 4^3,4^ while solid tumors are more acidic (pH 6.2–7.0) than normal tissue (pH ∼ 7.5).^5,6^ Very low pH is also observed during cellular studies of lysosomes 4.0–5.0.^7,8^ The procedures used for pH measurements in medicine rely on microelectrodes and several imaging techniques such as electron paramagnetic resonance (EPR), positron emission tomography (PET), photoacoustic imaging (PAI), and magnetic resonance imaging (MRI).^9^

MRI is a noninvasive diagnostic tool for soft tissues that uses the magnetic properties of the ^1^H nucleus. It is one of the most widely used imaging procedures in medicine and offers excellent spatial resolution and unlimited penetration depth and provides knowledge that cannot be accessed by other means.^10,11^ In order to improve the quality of the images, ^19^F MRI has been extensively investigated as a complementary modality that allows so-called hot spot imaging.^12,13^ Since ^19^F atoms are not present in soft tissues, this modality allows for the accurate representation of the targeted organ without background signals; however, it requires the introduction of an appropriate contrast agent containing ^19^F nuclei into the organism.^14^

Regarding the ^19^F NMR or MRI as an aid in pH measurements, several molecular probes have been developed. The earliest reports concerned fluorinated aniline derivatives, whose mechanism of action was based on protonation of the aniline nitrogen, which changed the chemical shift of the fluorine atom.^15−17^ Other concepts utilized PEGylated nanogels containing perfluorocarbons,^18^ C_6_F_6_-loaded Au-fluorescent mesoporous silica nanoparticles,^19^ or copolymers.^20−23^ In those cases, the mechanism of pH-depended signal changes was either based on the reversible volume phase transition of the nanogel, which emitted an ^19^F NMR signal, or the irreversible decomposition of the capsule under the influence of pH and the release of fluoroorganic groups, respectively.^24^ In each case, a single ^19^F signal with a pH-dependent chemical shift was observed. A probe displaying two separate signals with pH-dependent integral ratios (allowing internal reference and ratiometric measurements) would be more convenient for diagnostic purposes.

Hydrazone-based molecular switches could be used in such a role. Molecular switches are usually defined as molecules that can reversibly transform between two (or more) thermodynamically stable states.^25,26^ Such compounds are sensitive to external stimuli such as light, pH, or electric current and include photochromic switches, host–guest switches, and rotaxanes.^27−29^ The main applications of molecular switches is expected to include molecular electronics (high-density data storage) or organic diodes.^30^ They have been also reported as chemosensors or fluorescence imaging agents.^31,32^

Here, we demonstrate the first application of ratiometric reversible hydrazone-based molecular switches as ^19^F NMR/MRI pH-responsive compounds based on chemical shift changes.

The structures (Figure 1) were derived from Aprahamian’s hydrazone switch.^33^ The synthesis of hydrazones **1**-*E*, **2**-*E*, and **3**-*E* involved coupling trifluoromethylanilines with ethyl pyridyl-acetate.^34^ Briefly, trifluoromethylaniline in EtOH was treated with concentrated HCl and then NaNO_2_ at 0 °C to give trifluoromethyl-1-benzenediazonium chloride. In a separate flask, ethyl-2-pyridyl-acetate was treated at 0 °C with sodium acetate in EtOH–H_2_O (8:1). These two solutions were combined and stirred at 0 °C for 1 h and then overnight at RT. The resultant reaction mixture was washed with methylene chloride, and the organic fraction was washed twice with saturated sodium bicarbonate solution and dried over magnesium sulfate. The crude product was subjected to column chromatography (SiO_2_, CH_2_Cl_2_–MeOH, 8:1) to give pure compounds as an orange solid (yields, 55–65%).

**Figure 1:**
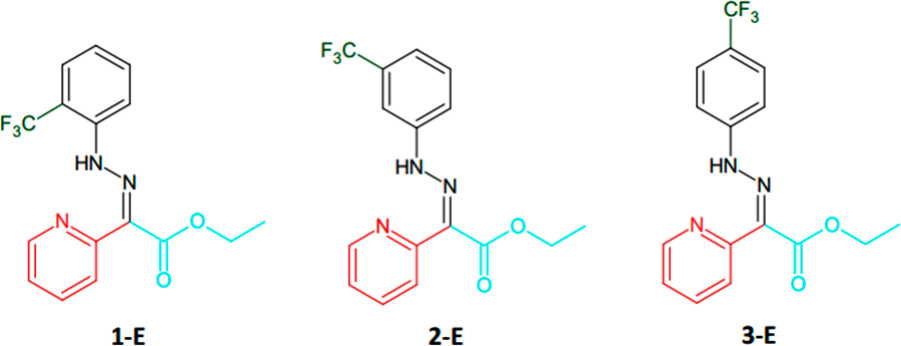
Chemical structures of fluorinated hydrazones.

According to X-ray crystallography, the structures were nearly planar, and H-bonding between the N–H proton and the pyridine nitrogen subunit was observed (Figure S13). The ^1^H NMR spectra of molecular switches (Figure 2) in CD_3_CN showed a characteristic H-bonded N–H resonance around 15 ppm, in addition to the expected aromatic and aliphatic signals.

**Figure 2:**
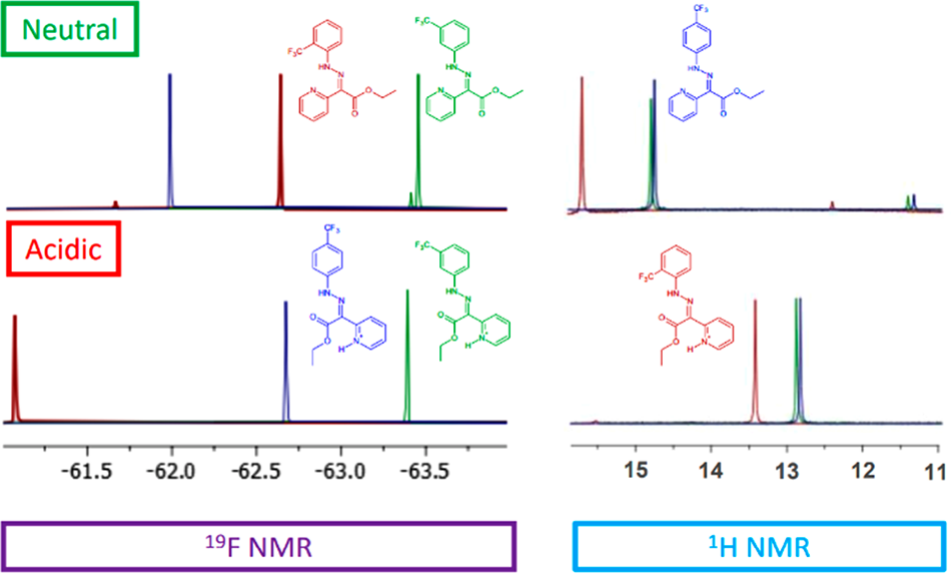
^19^F NMR (left) and ^1^H NMR (right) spectra (400 MHz) in CD_3_CN; (top) **1**-*E* (red), **2**-*E* (green), and **3**-*E* (blue); (bottom) *Z*-H^+^ recorded after the addition of 1.6 equiv of trifluoroacetic acid (TFA).

The N–H chemical shift indicates that the pyridine nitrogen is H-bonded with the N–H proton. A careful look at the ^1^H NMR spectrum shows a small signal near 12 ppm stemming from the minor *Z* configuration. The calculated geometries (B3LYP/6-31G(d, p) level of theory) of the two configurations in CD_3_CN showed that the *E* configuration is more stable than *Z*, which is in agreement with the isomer ratio observed in the ^1^H NMR spectrum. The addition of 1.6 equiv of TFA to CD_3_CN solutions of **1**-*E*, **2**-*E*, and **3**-*E* protonated the pyridine subunit, which was accompanied by a color change in the solution from light yellow to orange (Figure 3c) and drastic changes in the ^1^H NMR and ^19^F NMR spectra (Figure 2).

**Figure 3:**
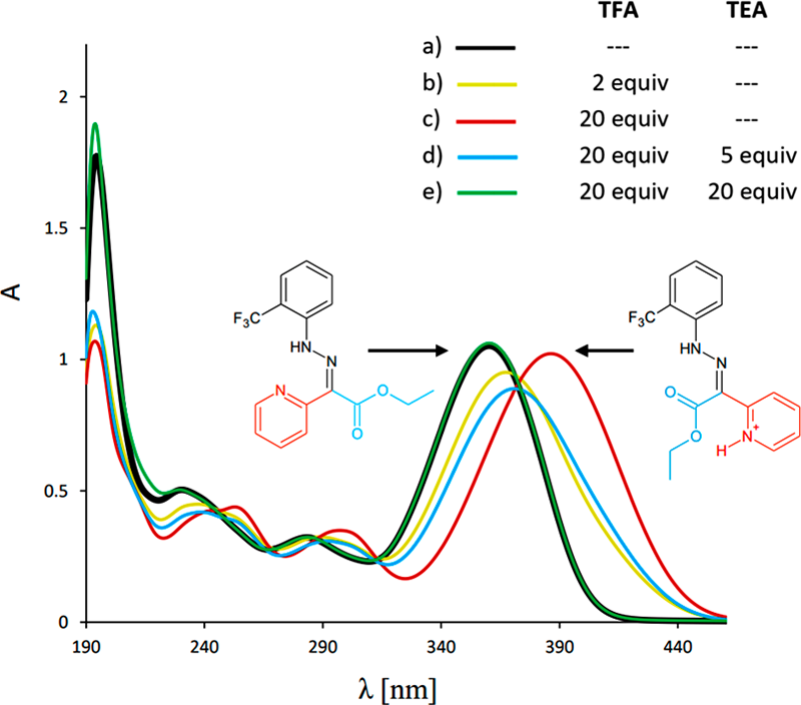
Changes in the UV–vis spectrum during the acid/base switching of **1**-*E*. All data were recorded in MeCN at 298 K using a 1.2 × 10^–4^ M solution of **1**-*E*. Spectra a and e are overlapping.

First, the N–H proton signals at 15 ppm disappeared, and new signals appeared at 13 ppm. This shift indicates that rotation around the C=N bond has occurred (*E*/*Z* isomerization) and that the N–H proton formed a hydrogen bond with the carbonyl group of the ester subunit, yielding *Z*-H^+^. Second, the pyridine proton signals shifted upfield, which is typical for protonated pyridine rings (Figure S9). In the ^19^F NMR spectra, peaks from the −CF_3_ group can be seen near −62.0 ppm. With the addition of TFA, these peaks moved toward higher frequencies for **1**-*E* and **3**-*E*, and those for the **2**-*E* peak moved toward a lower frequency. For the *o*-isomer (**1**-*E*), the greatest change in the chemical shift was −1.8 ppm. The ^19^F NMR signals were sharper (8 Hz) than the N–H signals recorded by ^1^H NMR (25 Hz) (width at half the peak height). To better describe the switching process, the *Z*-H^+^ molar fraction was calculated (based on peak integrals) and plotted against the pH of the solution (Figure 4). It can be seen that the switching process occurred between pH 4 and 3, and the range is practically identical for all isomers. The relationship from Figure 4 can be used for determination of the pH of a solution. Within the 3–4 range, it is possible with a standard uncertainty of 0.05 pH units. Outside this range, the pH can be estimated as either >4 or <3.

**Figure 4:**
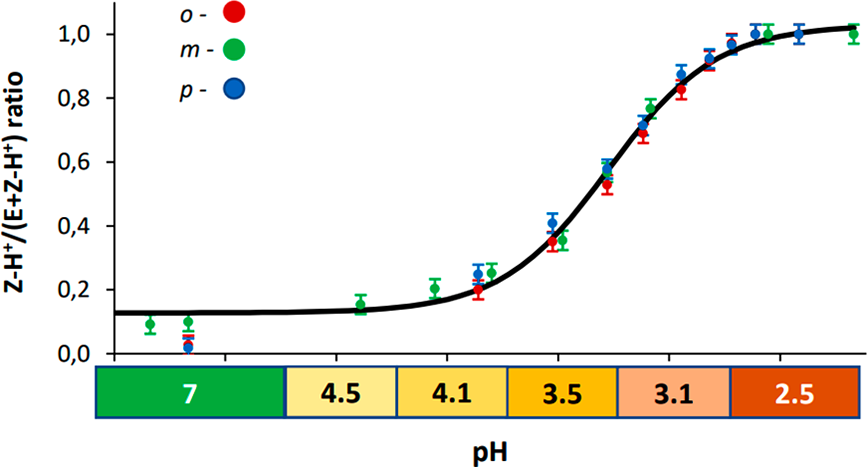
Representation of the pH-dependent switching process. Error bars represents standard uncertainy assuming 5% accuracy of the peak integration.

Upon the addition of 1.6 equiv of triethylamine (TEA) to the CD_3_CN solution of *Z*-H^+^, the color of the solution changed back to light yellow (Figure 3e). The ^1^H NMR and ^19^F NMR spectra (Figures S9–S11), recorded immediately after the addition of TEA, showed the complete disappearance of *Z*-H^+^ and the presence of both **1**-*E* and **1**-*Z* in solution. This is evident from the disappearance of the H-bonded N–H peak at 13 ppm and the appearance of two H-bonded N–H peaks (a larger one at 15 ppm and a smaller one at 12 ppm), which were assigned to *E* and *Z*, respectively. In each case, the *T*_1_ and *T*_2_ relaxation times of the fluorine atom of the *E*- and *Z*-H^+^ isomers were 1.4–1.6 s and did not change significantly during acidification (Figures S16–S18). Such behavior greatly simplifies the imaging procedure, as only variations of chemical shifts must be taken into account. Since the pH-dependent chemical shift changes are relatively large, the compounds can be used in the MRI of pH gradients. Figure 5 shows the ^19^F MRI images of acetonitrile solutions (15 mM) of **1**, which display the highest impact of pH on the chemical shift of the CF_3_ functionality. The images were acquired with the appropriate RF excitation pulse frequency and bandwidth of 0.5 ppm to cover the resonance frequencies of the isomers (Figure 5C,E,I). The required acquisition times were 15 min and only 2 min for FLASH and RARE sequences, respectively. We were also able to perform analogous imaging with compound **3** (Figure S15). Unfortunately, the difference in chemical shifts for the isomers of compound **2** (0.1 ppm) turned out to be too small to register good-quality images within a reasonable time. While the chemical shift of the *Z* isomer showed a certain pH dependence (Figures S9–S11), the spectral distance between the ^19^F lines of the *E* and *Z* isomers was large enough to obtain separate images for the entire range of pH values. If necessary, it is possible to increase the RF pulse bandwidth for MR imaging to determine the presence of the *Z* isomer and cover its entire pH-dependent range of chemical shifts.

**Figure 5:**
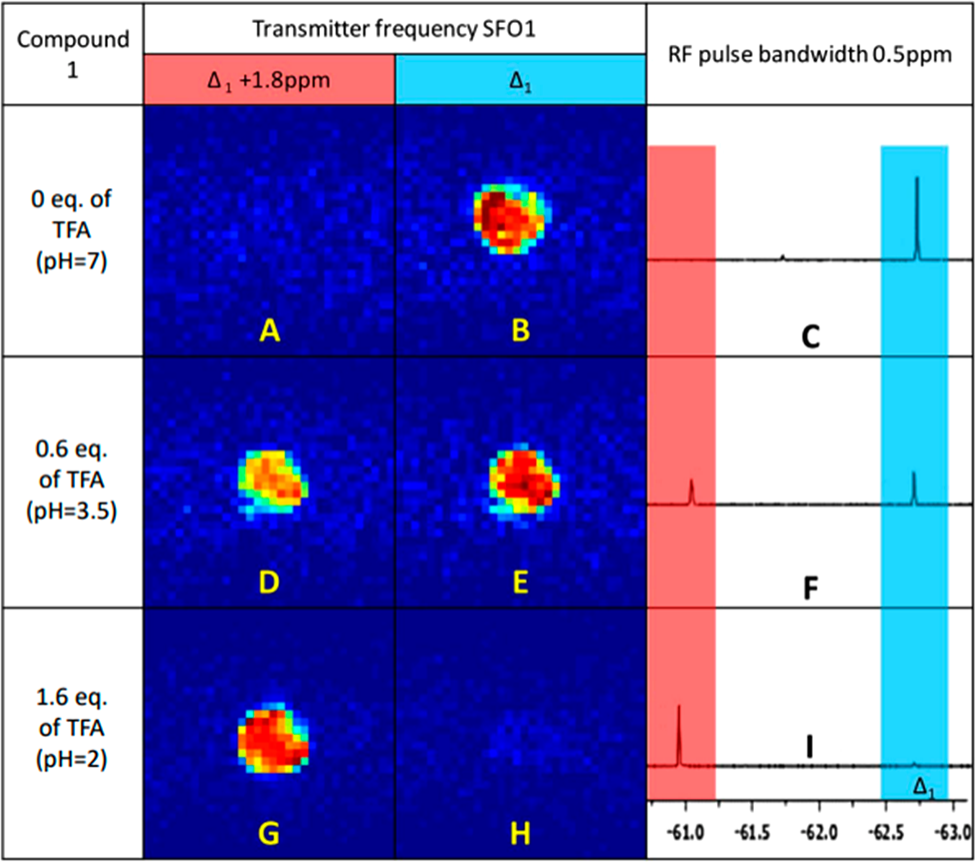
^19^F MRI of compound **1** in CH_3_CN. (A,D,G) RF excitation pulse at −60.9 ppm with 0.5 ppm bandwidth; (B,E,H) RF excitation pulse at −62.7 ppm with 0.5 ppm bandwidth; (C,F,I) corresponding ^19^F NMR spectra.

To explain the differences in the ^19^F NMR chemical shifts for the isomers **1**-*E*, **2**-*E*, and **3**-*E*, we performed DFT calculations to visualize their molecular orbitals. The shape of the orbitals clearly indicates an increased density of electrons in the *o*- and *p*-positions (Table S2), while a much lower density was observed at the *m*-positions. This is due to a mesomeric effect in which the nitrogen of the hydrazone group is an electron donor to the aromatic ring. The distinct range of changes in chemical shifts in the ^19^F NMR spectrum during *E*/*Z* configuration switching in compounds **1**–**3** can be explained based on the differences in the binding energies of the hydrazone hydrogen. For the *E* configuration, the bond was stronger (N–H---N), and for the *Z* configuration, it was weaker (N–H---O).^35^ Thus, the stronger bond polarization in the *E* configuration increased the electron-donating properties of the pyridyl nitrogen, which increased the electron density at the *o*- and *p*-positions. The highest change in the chemical shift was coupled with the biggest change in energy during switching for the *o*-isomer, as demonstrated in Figure 6. Additionally, the −CF_3_ group at the *o*-position was surrounded by strongly electronegative atoms (oxygen or nitrogen), which significantly impacted the magnetic properties of fluorine atoms.

**Figure 6:**
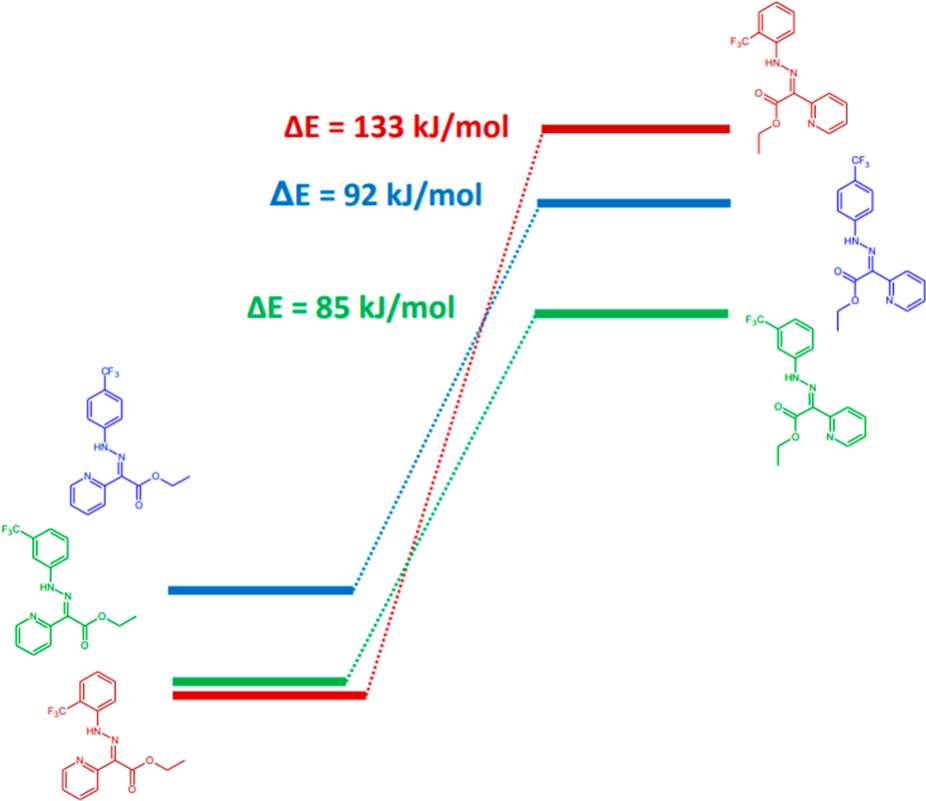
Calculated energy diagram for *E*/*Z* configuration changes of compounds **1**, **2**, and **3**.

Although the *E* isomers showed poor water solubility, they were highly soluble (>20–40 mg/mL) in polar organic solvents, and their protonated forms (*Z*-H^+^ isomers) were slightly soluble in water (Table S5). In practical applications for ^19^F MRI in medical diagnostics, insoluble fluoroorganic compounds are typically used as aqueous emulsions.^36^ In the case of **1**, we prepared a stable vegetable oil–water emulsion with pluronic-127 as a surfactant. The emulsion showed similar features in ^19^F NMR and ^19^F MRI experiments as the acetonitrile solution, except for higher peak widths of 80 Hz compared with 8 Hz in solution (Figures S19 and S21), which are still comparable to the RF pulse bandwidths used in previous MRI experiments.

In conclusion, we obtained a series of hydrazone molecular switches containing the −CF_3_ functionality. This allowed the visualization of the switching process by ^19^F NMR, ^1^H NMR, UV–vis, and ^19^F MRI in the low (millimolar) concentration range with a short acquisition time in both polar organic solvents or aqueous emulsions. The chemical shift changes for the **1**-*E* isomer were surprisingly large, which opens possibilities for applications of such molecular switches as smart MRI contrast agents. Further research is currently in progress in our laboratory to tune the pH-switching range and improve their aqueous solubility. The latter should be achieved by the introduction of polyethylene glycol or carbohydrate functionalities.^37^

## Supporting Information Available

Chemicals and reagents, measurements, hardware and software configuration, sample preparation, additional experimental details, materials, and methods (PDF)

## Dedication

We thank the Silesian University of Technology for the financial support (Grant 04/050/BKM21/0132)

## References

[ref1] Di Costanzo L.; Panunzi B. Visual PH Sensors: From a Chemical Perspective to New Bioengineered Materials. Molecules 2021, 26 (10), 2952 10.3390/molecules26102952. 34065629 PMC8156760

[ref2] Monteiro M. C. O.; Koper M. T. M. Measuring Local pH in Electrochemistry. Curr. Opin. Electrochem. 2021, 25, 100649 10.1016/j.coelec.2020.100649.

[ref3] Benitz W. Infectious Disease and Pharmacology; Elsevier, 2019.

[ref4] Badillo R. Diagnosis and Treatment of Gastroesophageal Reflux Disease. World J. Gastrointest. Pharmacol. Ther. 2014, 5 (3), 105 10.4292/wjgpt.v5.i3.105. 25133039 PMC4133436

[ref5] Tannock I. F.; Rotin D. Acid pH in Tumors and Its Potential for Therapeutic Exploitation. Cancer Res. 1989, 49 (16), 4373–4384. 2545340

[ref6] Hao G.; Xu Z. P.; Li L. Manipulating Extracellular Tumour pH: An Effective Target for Cancer Therapy. RSC Adv. 2018, 8 (39), 22182–22192. 10.1039/C8RA02095G. PMC9081285 35541713

[ref7] Li S.-S.; Zhang M.; Wang J.-H.; Yang F.; Kang B.; Xu J.-J.; Chen H.-Y. Monitoring the Changes of pH in Lysosomes during Autophagy and Apoptosis by Plasmon Enhanced Raman Imaging. Anal. Chem. 2019, 91 (13), 8398–8405. 10.1021/acs.analchem.9b01250. 31144810

[ref8] Zeng J.; Shirihai O. S.; Grinstaff M. W. Modulating Lysosomal pH: A Molecular and Nanoscale Materials Design Perspective. JoLS, J. Life Sci. 2020, 2 (4), 25–37. 10.36069/JoLS/20201204. PMC7781074 33403369

[ref9] Anemone A.; Consolino L.; Arena F.; Capozza M.; Longo D. L. Imaging Tumor Acidosis: A Survey of the Available Techniques for Mapping in Vivo Tumor pH. Cancer Metastasis Rev. 2019, 38 (1–2), 25–49. 10.1007/s10555-019-09782-9. 30762162 PMC6647493

[ref10] Pierre V. C.; Allen M. J.; Caravan P. Contrast Agents for MRI: 30+ Years and Where Are We Going?. JBIC J. Biol. Inorg. Chem. 2014, 19 (2), 127–131. 10.1007/s00775-013-1074-5. 24414380 PMC4075138

[ref11] Wahsner J.; Gale E. M.; Rodríguez-Rodríguez A.; Caravan P. Chemistry of MRI Contrast Agents: Current Challenges and New Frontiers. Chem. Rev. 2019, 119 (2), 957 10.1021/acs.chemrev.8b00363. 30350585 PMC6516866

[ref12] Hequet E.; Henoumont C.; Muller R. N.; Laurent S. Fluorinated MRI Contrast Agents and Their Versatile Applications in the Biomedical Field. Future Med. Chem. 2019, 11 (10), 1157–1175. 10.4155/fmc-2018-0463. 31280670

[ref13] Janasik D.; Krawczyk T. 19F MRI Probes for Multimodal Imaging**. Chem. Eur. J. 2022, 28 (5), e202102556 10.1002/chem.202102556. 34705306

[ref14] Peterson K. L.; Srivastava K.; Pierre V. C. Fluorinated Paramagnetic Complexes: Sensitive and Responsive Probes for Magnetic Resonance Spectroscopy and Imaging. Front. Chem. 2018, 10.3389/fchem.2018.00160. PMC5974164 29876342

[ref15] Deutsch C. J.; Taylor J. S. Intracellular pH as Measured by 19F NMR. Ann. N.Y. Acad. Sci. 1987, 508, 33–47. 10.1111/j.1749-6632.1987.tb32892.x. 3501935

[ref16] Frenzel T.; Koler S.; Bauer H.; Niedballa U.; Weinmann H. J. Noninvasive in Vivo pH Measurements Using a Fluorinated pH Probe and Fluorine-19 Magnetic Resonance Spectroscopy. Investigative Radiology 1994, 29, S220–S222. 10.1097/00004424-199406001-00073. 7928237

[ref17] Metcalfe J. C.; Hesketh T. R.; Smith G. A. Free Cytosolic Ca^2+^ Measurements with Fluorine Labelled Indicators Using 19F NMR. Cell Calcium 1985, 6 (1–2), 183–195. 10.1016/0143-4160(85)90043-0. 3874697

[ref18] Oishi M.; Sumitani S.; Bronich T. K.; Kabanov A. V.; Boska M. D.; Nagasaki Y. Novel 19F MRS/I Nanoprobe Based on pH-Responsive PEGylated Nanogel: pH-Dependent 19F Magnetic Resonance Studies. Chem. Lett. 2009, 38 (2), 128–129. 10.1246/cl.2009.128.

[ref19] Chen S.; Yang Y.; Li H.; Zhou X.; Liu M. PH-Triggered Au-Fluorescent Mesoporous Silica Nanoparticles for 19F MR/Fluorescent Multimodal Cancer Cellular Imaging. Chem. Commun. 2014, 50 (3), 283–285. 10.1039/C3CC47324D. 24170041

[ref20] Huang X.; Huang G.; Zhang S.; Sagiyama K.; Togao O.; Ma X.; Wang Y.; Li Y.; Soesbe T. C.; Sumer B. D.; Takahashi M.; Sherry A. D.; Gao J. Multi-Chromatic pH-Activatable 19F-MRI Nanoprobes with Binary ON/OFF pH Transitions and Chemical-Shift Barcodes. Angew. Chemie Int. Ed. 2013, 52 (31), 8074–8078. 10.1002/anie.201301135. PMC3771347 23788453

[ref21] Li Y.; Zhang H.; Guo C.; Hu G.; Wang L. Multiresponsive Nanoprobes for Turn-On Fluorescence/19F MRI Dual-Modal Imaging. Anal. Chem. 2020, 92 (17), 11739–11746. 10.1021/acs.analchem.0c01786. 32786481

[ref22] Zhang C.; Li L.; Han F. Y.; Yu X.; Tan X.; Fu C.; Xu Z. P.; Whittaker A. K. Integrating Fluorinated Polymer and Manganese-Layered Double Hydroxide Nanoparticles as pH-Activated 19F MRI Agents for Specific and Sensitive Detection of Breast Cancer. Small 2019, 15 (36), 1902309 10.1002/smll.201902309. 31328398

[ref23] Gianolio E.; Napolitano R.; Fedeli F.; Arena F.; Aime S. Poly-β-Cyclodextrin Based Platform for pH Mapping via a Ratiometric 19F/1H MRI Method. Chem. Commun. 2009, 40, 6044 10.1039/b914540k. 19809638

[ref24] Bonnet C. S.; Tóth É. Smart Contrast Agents for Magnetic Resonance Imaging. Chim. Int. J. Chem. 2016, 70 (1), 102–108. 10.2533/chimia.2016.102. 26931225

[ref25] Aprahamian I. Hydrazone Switches and Things in Between. Chem. Commun. 2017, 53 (50), 6674–6684. 10.1039/C7CC02879B. 28540954

[ref26] Harris J. D.; Moran M. J.; Aprahamian I. New Molecular Switch Architectures. Proc. Natl. Acad. Sci. U. S. A. 2018, 115 (38), 9414–9422. 10.1073/pnas.1714499115. 30012601 PMC6156620

[ref27] Feringa B. L. Molecular Switches; Wiley VCH, 2001.

[ref28] Nacci C.; Baroncini M.; Credi A.; Grill L. Reversible Photoswitching and Isomer-Dependent Diffusion of Single Azobenzene Tetramers on a Metal Surface. Angew. Chemie Int. Ed. 2018, 57 (46), 15034–15039. 10.1002/anie.201806536. PMC6237119 30187995

[ref29] Arramel; Pijper T. C.; Kudernac T.; Katsonis N.; van der Maas M.; Feringa B. L.; van Wees B. J. Reversible Light Induced Conductance Switching of Asymmetric Diarylethenes on Gold: Surface and Electronic Studies. Nanoscale 2013, 5 (19), 9277 10.1039/c3nr00832k. 24163831

[ref30] Jerca V. V.; Hoogenboom R. Photocontrol in Complex Polymeric Materials: Fact or Illusion?. Angew. Chemie Int. Ed. 2018, 57 (27), 7945–7947. 10.1002/anie.201804027. 29863783

[ref31] Wu D.; Sedgwick A. C.; Gunnlaugsson T.; Akkaya E. U.; Yoon J.; James T. D. Fluorescent Chemosensors: The Past, Present and Future. Chem. Soc. Rev. 2017, 46 (23), 7105–7123. 10.1039/C7CS00240H. 29019488

[ref32] Xia T.; Li N.; Fang X. Single-Molecule Fluorescence Imaging in Living Cells. Annu. Rev. Phys. Chem. 2013, 64 (1), 459–480. 10.1146/annurev-physchem-040412-110127. 23331306

[ref33] Landge S. M.; Tkatchouk E.; Benítez D.; Lanfranchi D. A.; Elhabiri M.; Goddard W. A.; Aprahamian I. Isomerization Mechanism in Hydrazone-Based Rotary Switches: Lateral Shift, Rotation, or Tautomerization?. J. Am. Chem. Soc. 2011, 133 (25), 9812–9823. 10.1021/ja200699v. 21585197

[ref34] Su X.; Lessing T.; Aprahamian I. The Importance of the Rotor in Hydrazone-Based Molecular Switches. Beilstein J. Org. Chem. 2012, 8, 872–876. 10.3762/bjoc.8.98. 23015836 PMC3388876

[ref35] Su X.; Lõkov M.; Kütt A.; Leito I.; Aprahamian I. Unusual Para-Substituent Effects on the Intramolecular Hydrogen-Bond in Hydrazone-Based Switches. Chem. Commun. 2012, 48 (85), 10490 10.1039/c2cc35860c. 22990382

[ref36] Wu L.; Liu F.; Liu S.; Xu X.; Liu Z.; Sun X. Perfluorocarbons-Based 19F Magnetic Resonance Imaging in Biomedicine. Int. J. Nanomedicine 2020, 15, 7377–7395. 10.2147/IJN.S255084. 33061385 PMC7537992

[ref37] Yu M.; Bouley B. S.; Xie D.; Que E. L. Highly Fluorinated Metal Complexes as Dual 19F and PARACEST Imaging Agents. Dalt. Trans. 2019, 48 (25), 9337–9341. 10.1039/C9DT01852B. PMC6626988 31168527

