# Supplementary material for: Ratiometric pH-Responsive 19F Magnetic Resonance Imaging Contrast Agents Based on Hydrazone Switches
Source: Anal Chem. 2022 Feb 14;94(8):3427–31. doi: 10.1021/acs.analchem.1c04978 (PMC8892427; doi:10.1021/acs.analchem.1c04978)
Supplement: Supplementary file 1 — ac1c04978_si_001.pdf [file ac1c04978_si_001.pdf]

Supporting Information

Ratiometric pH-responsive ^{19}F MRI contrast agents based on hydrazone switches

Dawid Janasik[a], Krzysztof Jasiński[b], Władysław P. Węglarz[b], Paweł Jewuła[c], Ivan Nemec[c,d], and Tomasz Krawczyk[a]*

[a] *Department of Chemical Organic Technology and Petrochemistry, Silesian University of Technology, Krzywoustego 4, 44-100 Gliwice, Poland, e-mail: tomasz.krawczyk@posl.pl*
[b] *Institute of Nuclear Physics Polish Academy of Sciences, 31-342 Krakow, Poland*
[c] *Central European Institute of Technology, Brno University of Technology, Purkyňova 123, 612-00 Brno, Czech Republic*
[d] *Department of Inorganic Chemistry, Faculty of Science, Palacký University, 17. Listopadu 12, 771-46 Olomouc, Czech Republic*

Table of contents

1. General S3

2. Synthesis S3

3. NMR spectra S5

4. UV-Vis spectra S11

5. Single Crystal Diffraction S11

6. Computational Methods S12

7. pH-dependent ^{19}F MRI S14

8. Relaxation times S17

9. Solubility S18

10. Emulsion S18

References S19

1. General

Unless otherwise noted, all reagents and starting materials were purchased from commercial vendors and used without further purification. All experiments were conducted in the air unless otherwise noted. Column chromatography was performed using silica gel (60 Å, 230-400 mesh). Mass analysis was performed by UPLC-MS-MS (Waters Xevo G2 QTof, ESI ionization, high-resolution TOF detection). NMR spectra were recorded on a 400 MHz Agilent spectrometer and referenced internally using the residual protonated solvent resonances relative to tetramethylsilane (δ= 0 ppm), or trifluoroacetic acid (^{19}F NMR, δ= -76.5 ppm). UV-Vis spectra were recorded using a JASCO V-650 UV-Vis spectrophotometer.

2. Synthesis

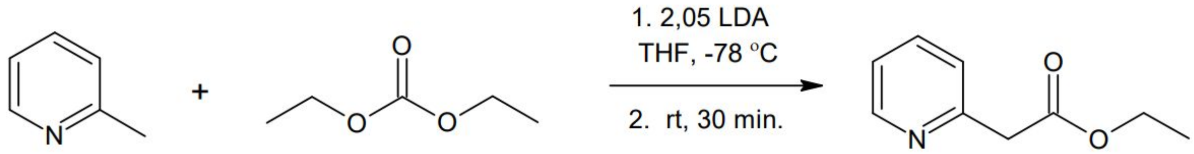

Ethyl 2-(pyridin-2-yl)acetate. *n*-BuLi (2.5 M solution in hexanes, 26.4 mL, 66 mmol, 2.05 eq.) was added dropwise to a stirred solution of diisopropylamine (6.8 g, 9.5 mL, 68 mmol, 2.10 eq.) in THF (40 mL) at −78 °C under Ar. The resulting solution was warmed to 0 °C and stirred at 0 °C for 1 h. Then, the solution was transferred to a stirred solution of 2-picoline (3.0 g, 3.2 mL, 32 mmol, 1.0 eq.) and diethyl carbonate (11.4 g, 11.7 mL, 97 mmol, 3.0 eq.) in THF (40 mL) at −78 °C under Ar. The resulting solution was stirred at −78 °C for 1 h and then allowed to warm to room temperature and stirred for 30 min. Saturated $NH_4Cl_{(aq.)}$ (20 mL) and water (50 mL) were added, and the two layers were separated, and the aqueous layer was extracted with Et_2O (3 × 30 mL). The combined organic layers were dried with $MgSO_4$ and evaporated under reduced pressure to give the product (4.5 g, 85%) as a bright yellow oil.

^1H NMR (400 MHz, $CDCl_3$, 298 K) δ = 8.55 (d, *J* = 7.0 Hz 1H, H10), 7.66 (t, *J* = 7,0 Hz, 1H, H12), 7.29 (d, *J* = 7.0 Hz, 1H, H13), 7.19 (dd, *J* = 7,0, 5,0 Hz, 1H, H11), 4,18 (q, *J* = 7.0 Hz, 2H, H17), 3,84 (s, 2H, H15), 1,26 (t, *J* = 7.0 Hz, 3H, H18); ^{13}C NMR (100 MHz, $CDCl_3$) δ 170.7 (C16), 154.5 (C14), 149,5 (C10), 136.7 (C12), 123.9 (C13), 122.1 (C11), 61.1 (C17), 40.0 (C15), 14.2 (C18); HR-MS (ESI): m/z calcd. for $C_9H_{12}NO_2$, 166.0868; found $[M-H]^+$: 166.0865.

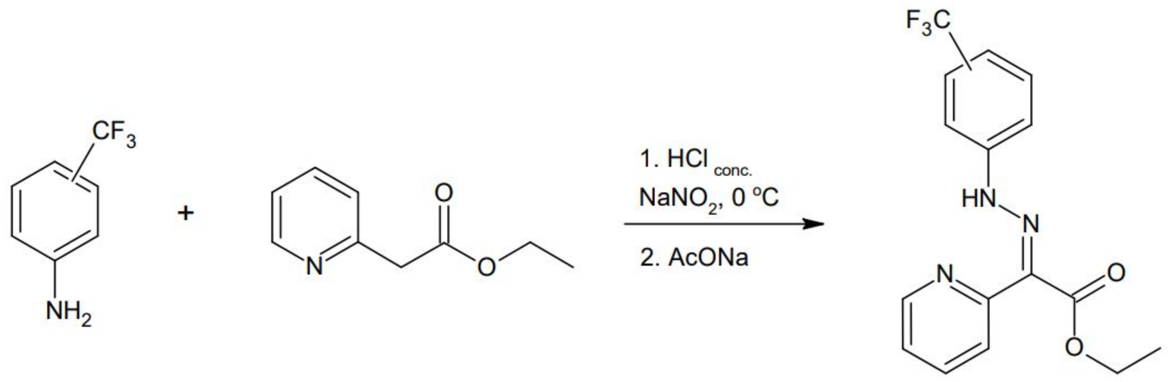

Compounds:

ethyl (2*E*)-(pyridin-2-yl) {2-[2-(trifluoromethyl)phenyl]hydrazinylidene}acetate (**1-*E***),

ethyl (2*E*)-(pyridin-2-yl) {2-[3-(trifluoromethyl)phenyl]hydrazinylidene}acetate (**2-*E***),

ethyl (2*E*)-(pyridin-2-yl) {2-[4-(trifluoromethyl)phenyl]hydrazinylidene}acetate (**3-*E***).

Trifluoromethylaniline (1.0 g, 6.2 mmol, 1 eq.) was dissolved in a mixture of 10.0 mL conc. HCl and 10.0 mL of EtOH and stirred in an ice bath for 30 min. A cold solution (8.0 mL) of sodium nitrite (0.4 g, 6.2 mmol, 1 eq.) was then added dropwise to the acidified solution over a period of 30 min. The obtained diazonium salt solution was then added dropwise to a suspension of ethyl-2-pyridylacetate (1.0 mL, 6.2 mmol, 1 eq.) and sodium acetate (3.3 g, 36.7 mmol, 6,4 eq.) in a cooled 40 mL ethanol/water (8: 1) mixture. The resultant reaction mixture was stirred overnight and then washed with methylene chloride. The organic fraction was washed twice with 30 mL saturated sodium bicarbonate solution and dried over magnesium sulfate. The crude product was then subjected to silica gel column chromatography (methanol/methylene chloride 1: 8) to give the pure compound as a bright orange solid.

1-*E* (Yield = 65%)

^{1}H NMR (400 MHz, CD_3CN, 298 K) δ = 15.54 (s, 1H, NH), 8.64 (ddd, *J* = 5.0, 2.0, 0.5 Hz, 1H), 8.14 (dt, *J* = 8.0, 2.0 Hz, 1H), 7.94 (m, 2H), 7.62 (m, 2H), 7.42 (dd, *J* = 8.0, 2.0 Hz, 1H), 7.12 (t, *J* = 8.0 Hz, 1H), 4.35 (q, *J* = 7.1 Hz, 2H), 1.39 (t, *J* =7.1 Hz, 3H); ^{13}C NMR (100 MHz, CD_3CN) δ 165.9, 152.4, 147.5, 142.2, 138.5, 137.5, 134.7, 130.2, 127.2 (q, J = 3.1 Hz), 127.0, 125.4, 124.6, 122.7, 116.1, 62.1, 14.6; HR-MS (ESI): m/z calcd. for $C_{16}H_{15}N_3O_2F_3$ 338.1116 [M-H]$^+$; found 338.1115. Melting point: 54.7-56.0 °C.

2-*E* (Yield = 63%)

^{1}H NMR (400 MHz, $CDCl_3$, 298 K) δ = 14.89 (s, 1H, NH), 8.66 (ddd, *J* = 5.0, 2.0, 0.5 Hz, 1H, H10), 8.23 (dt, *J* = 8.0, 2.0 Hz, 1H, H12), 7.83 (td, *J* = 7.5, 1.5 Hz, 1H, H13), 7.58 (s, 1H, H7), 7.54 (d, *J* = 8.0 Hz, 1H, H3), 7.44 (t, *J* = 8.0 Hz, 1H, H4), 7.30 (ddd, *J* = 7.9, 2.1, 0.5 Hz, 1H, H11), 7.25 (d, *J* = 7.9 Hz, 1H, H5), 4.40 (q, *J* = 7.1 Hz, 2H, H17), 1.45 (t, *J* =7.1 Hz, 3H, H18); ^{13}C NMR (100 MHz, $CDCl_3$) δ 165.4 (C16), 152.4

(C15), 146.5 (C14), 143.9 (C6), 136.9 (C10), 131.5 (q, J = 3.1 Hz, C1), 129.8 (C12), 126.9 (C2), 124.5 (C4), 123.4 (C11), 122.7 (C13), 118.9 (C3), 117.7 (C5), 111.5 (C7), 61.2 (C17), 14.3 (C18); HR-MS (ESI): m/z calcd. for $C_{16}H_{15}N_3O_2F_3$ 338.1116 [M-H]$^+$; found 338.1119. Melting point: 76.8-77.5 °C.

3-*E* (Yield = 55%)

^{1}H NMR (400 MHz, $CDCl_3$, 298 K) δ = 14.85 (s, 1H, NH), 8.64 (ddd, J = 5.0, 2.0, 0.5 Hz, 1H), 8.19 (dt, J = 8.0, 2.0 Hz, 1H), 7.83 (td, J = 7.5, 1.5 Hz 1H), 7.57 (d, J = 8Hz 2H), 7.42 (d, J = 8Hz 2H), 7.30 (dd, J = 8.0, 2.0 Hz, 1H), 4.40 (q, J = 7.1 Hz, 2H), 1.45 (t, J =7.1 Hz, 3H); ^{13}C NMR (100 MHz, $CDCl_3$) δ 165.4, 152.2, 148.7, 146.5, 146.0, 137.0, 136.2, 127.5, 126,6 (q, J = 3.1 Hz),124.6, 123.4, 122.7, 122.7, 114.4, 114.0, 61.2, 14.3; HR-MS (ESI): m/z calcd. for $C_{16}H_{15}N_3O_2F_3$ 338.1116 [M-H]$^+$; found 338.1112. Melting point: 67.5-67.7 °C.

3. NMR spectra

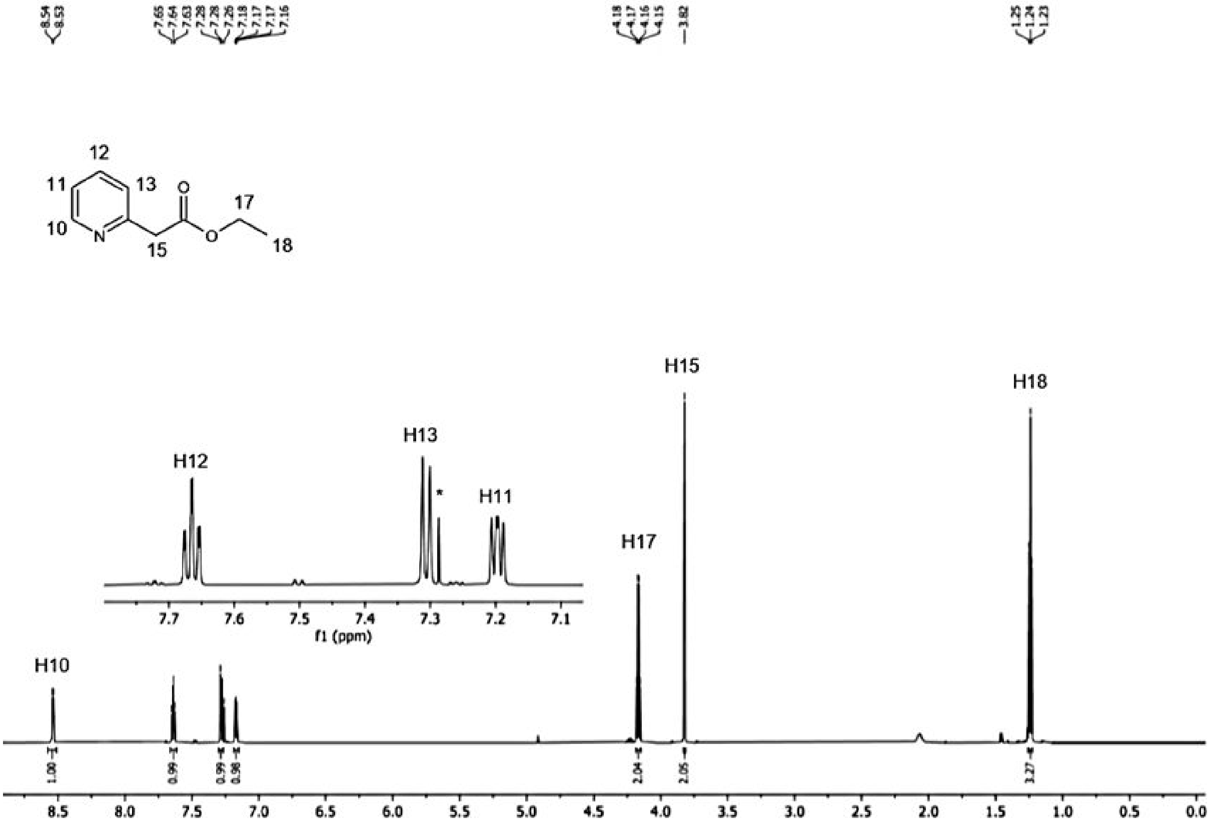

Figure S1. ^{1}H NMR spectrum of the ethyl 2-(pyridin-2-yl)acetate. $CDCl_3$, 400 MHz, T = 298 K.

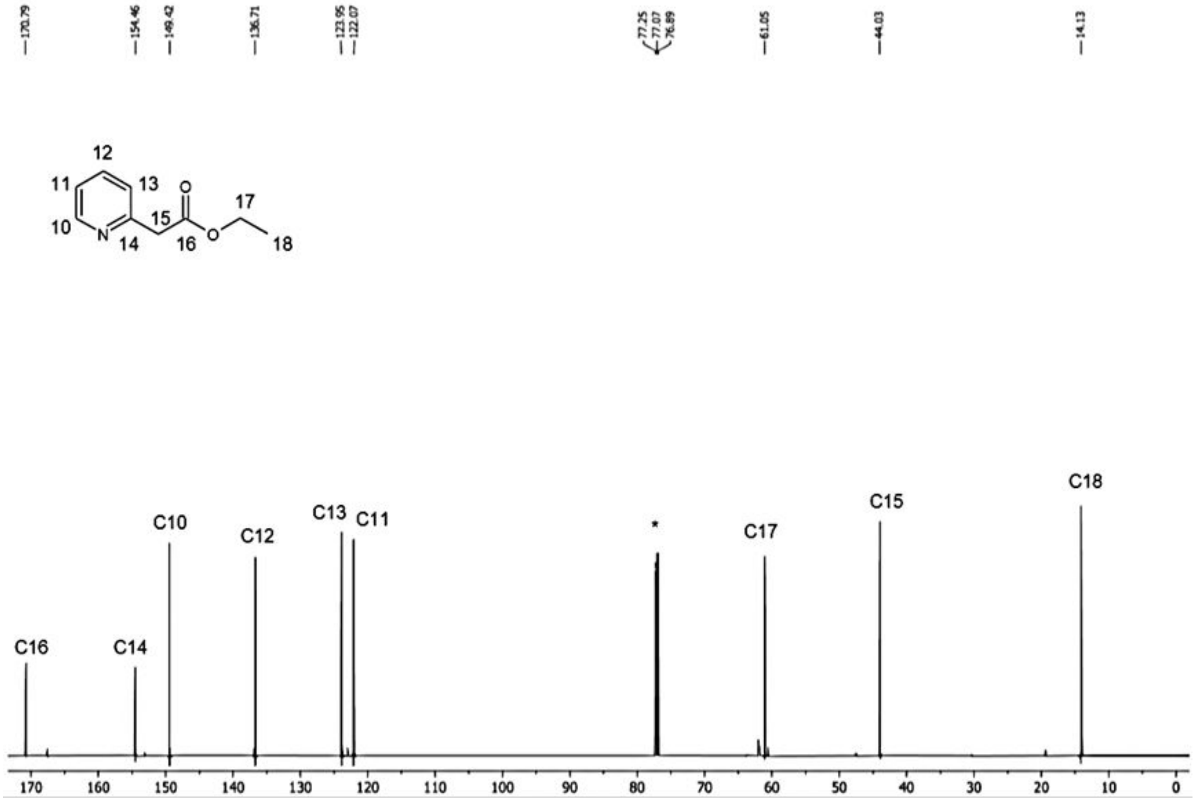

Figure S2. ^{13}C spectrum of the ethyl 2-(pyridin-2-yl)acetate. $CDCl_3$, 100 MHz, *T* = 298 K.

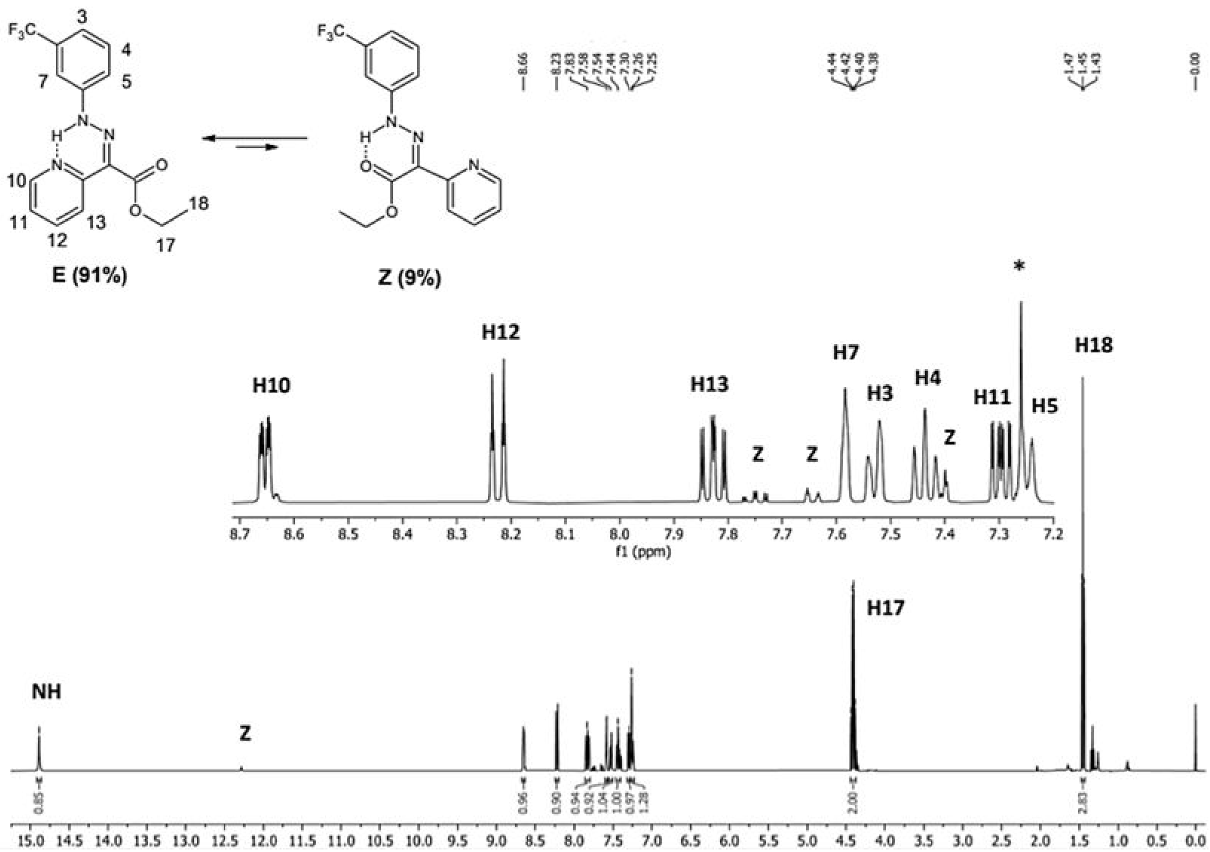

Figure S3. ^{1}H NMR spectrum of 91% **2-*E*** and 9% **2-*Z***. $CDCl_3$, 400 MHz, *T* = 298 K.

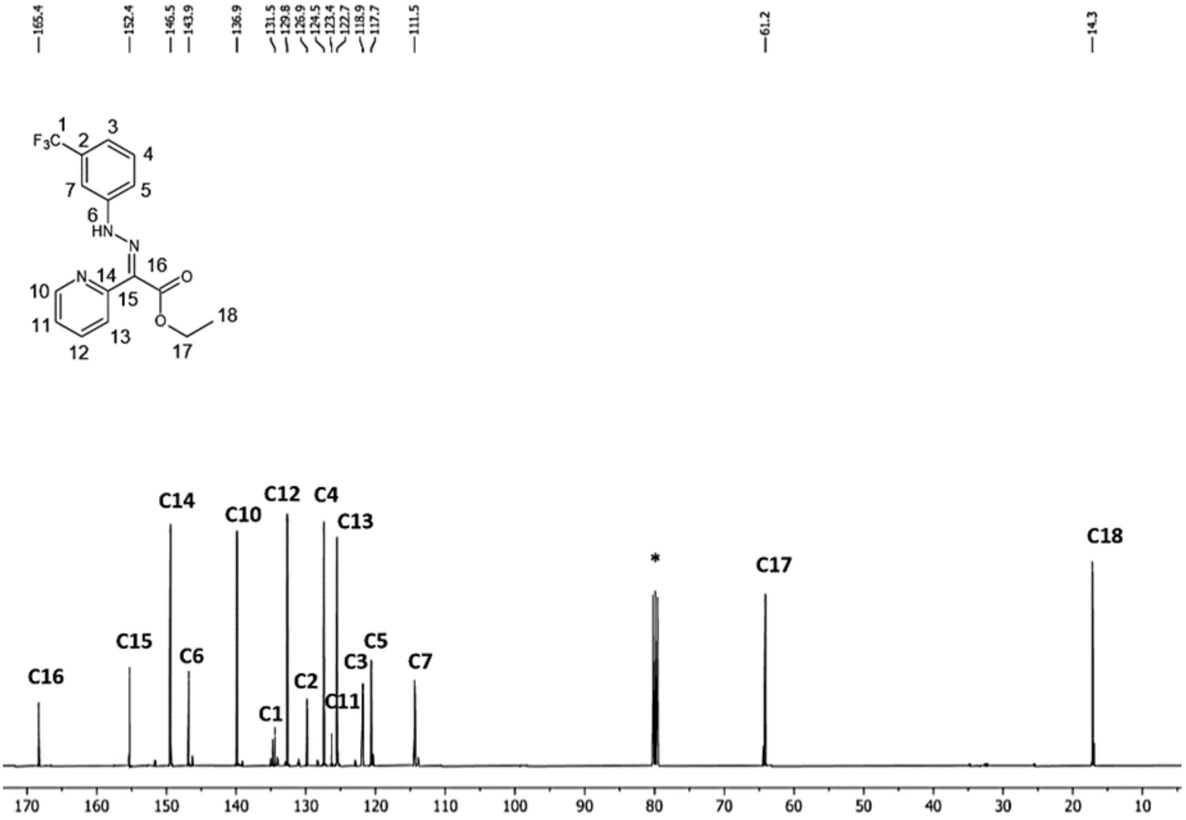

Figure S4. ^{13}C spectrum of the **2-*E***. $CDCl_3$, 100 MHz, *T* = 298 K.

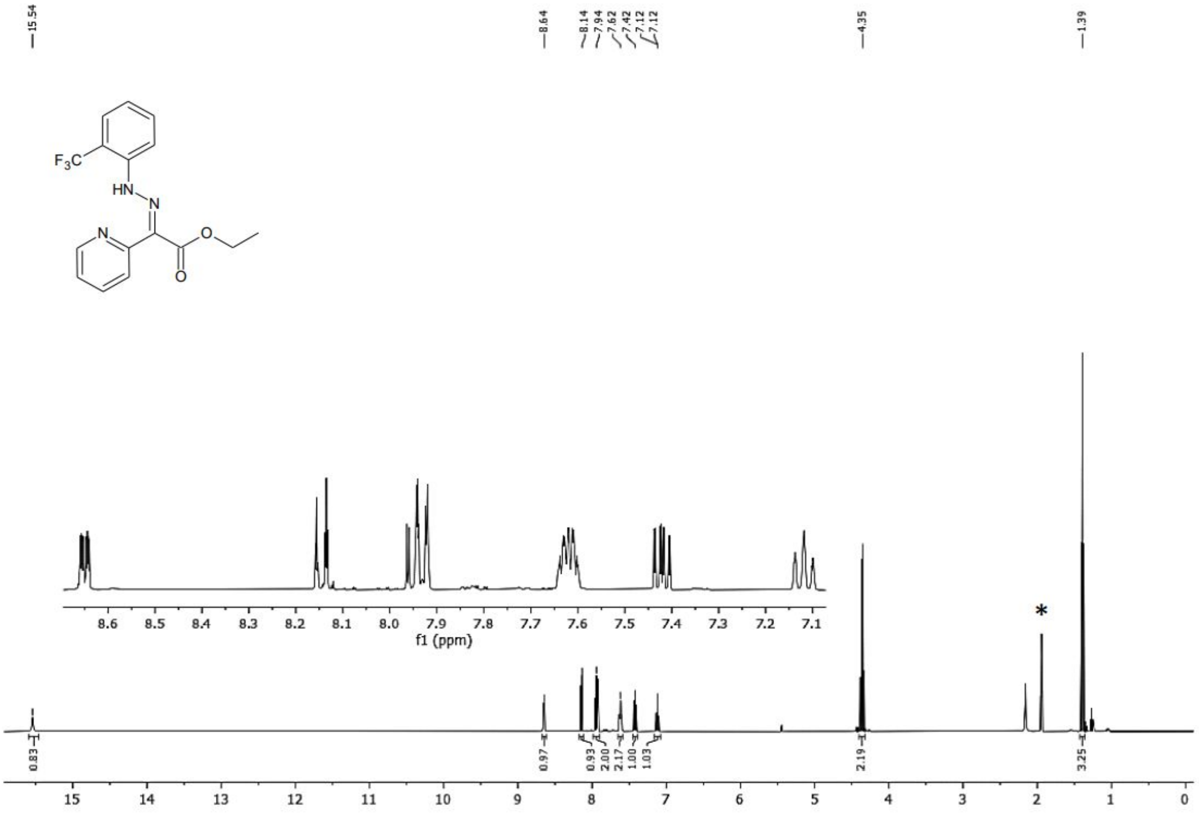

Figure S5. ^{1}H NMR spectrum of **1-*E***. CD_3CN, 400 MHz, *T* = 298 K.

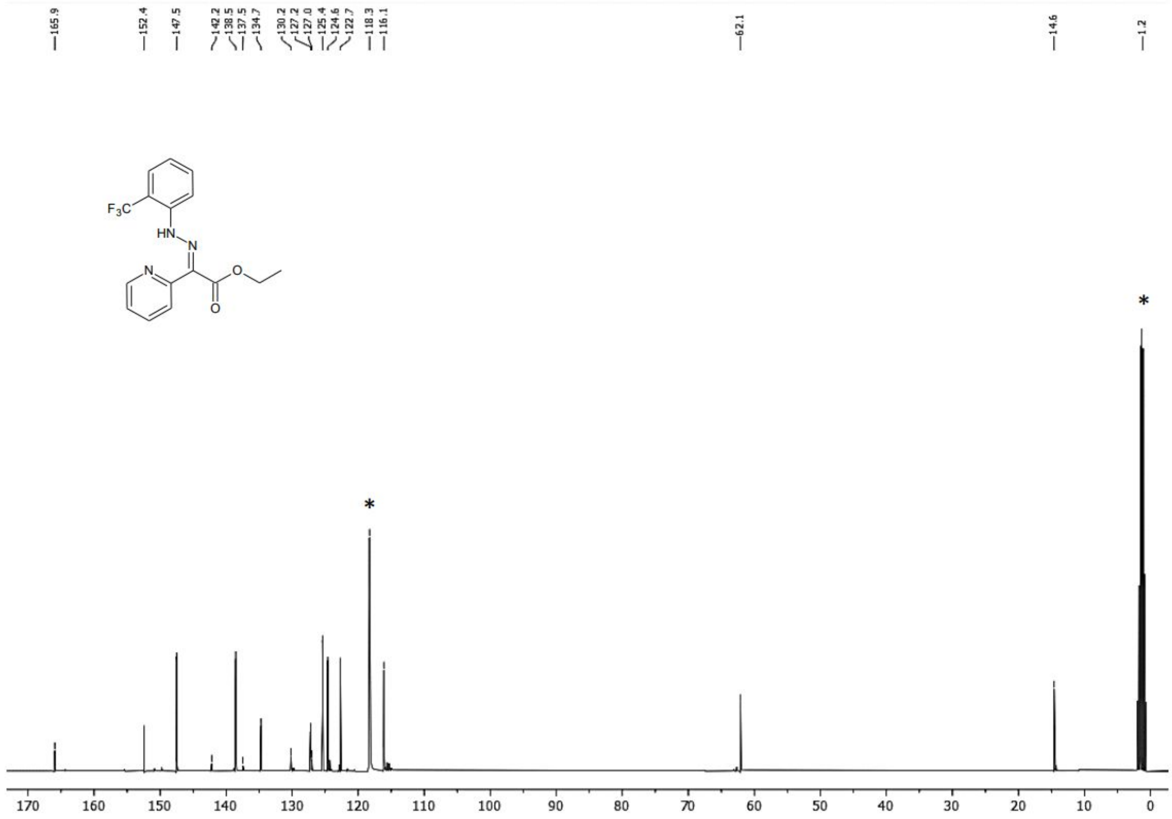

Figure S6. ^{13}C spectrum of the ***1-E***. CD_3CN, 100 MHz, T = 298 K.

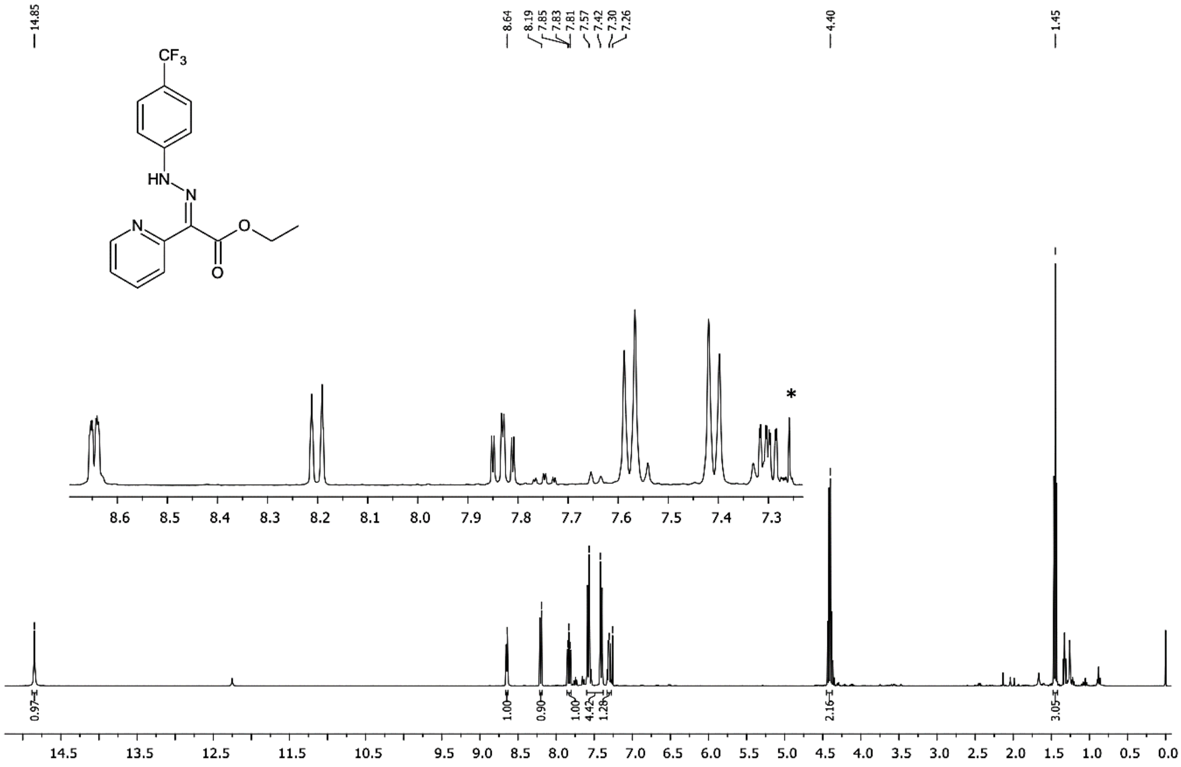

Figure S7. ^{1}H NMR spectrum of 94% ***3-E*** and 6% ***3-Z***. $CDCl_3$, 400 MHz, T = 298 K.

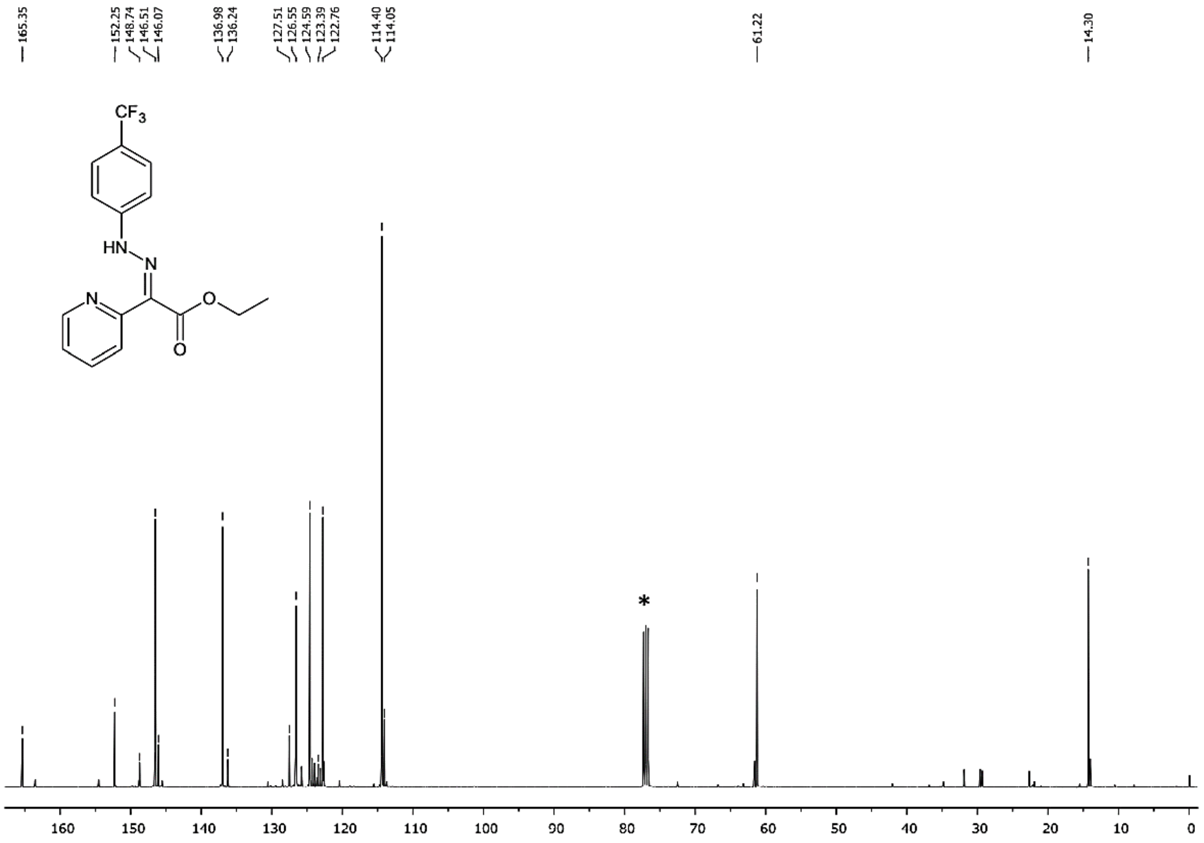

Figure S8. ^{13}C spectrum of the **3-*E*.** $CDCl_3$, 100 MHz, T = 298 K.

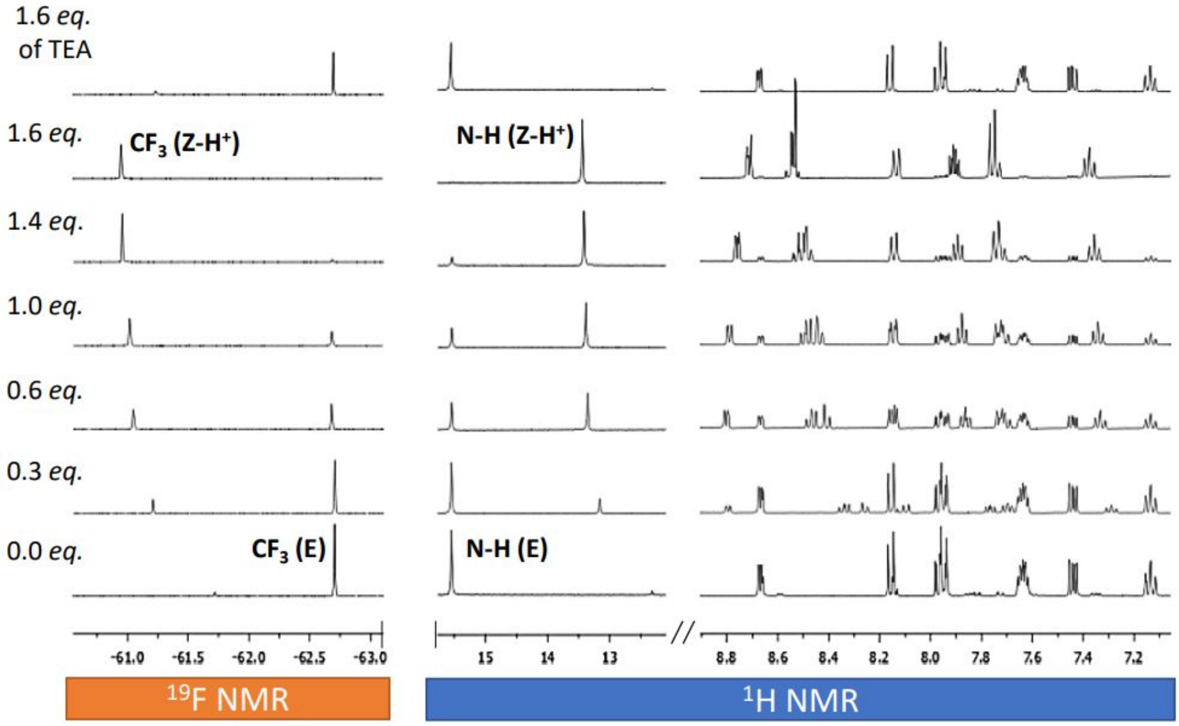

Figure S9. Acid/base switching monitored by ^{19}F and ^{1}H NMR for **1-*E*,** CD_3CN.

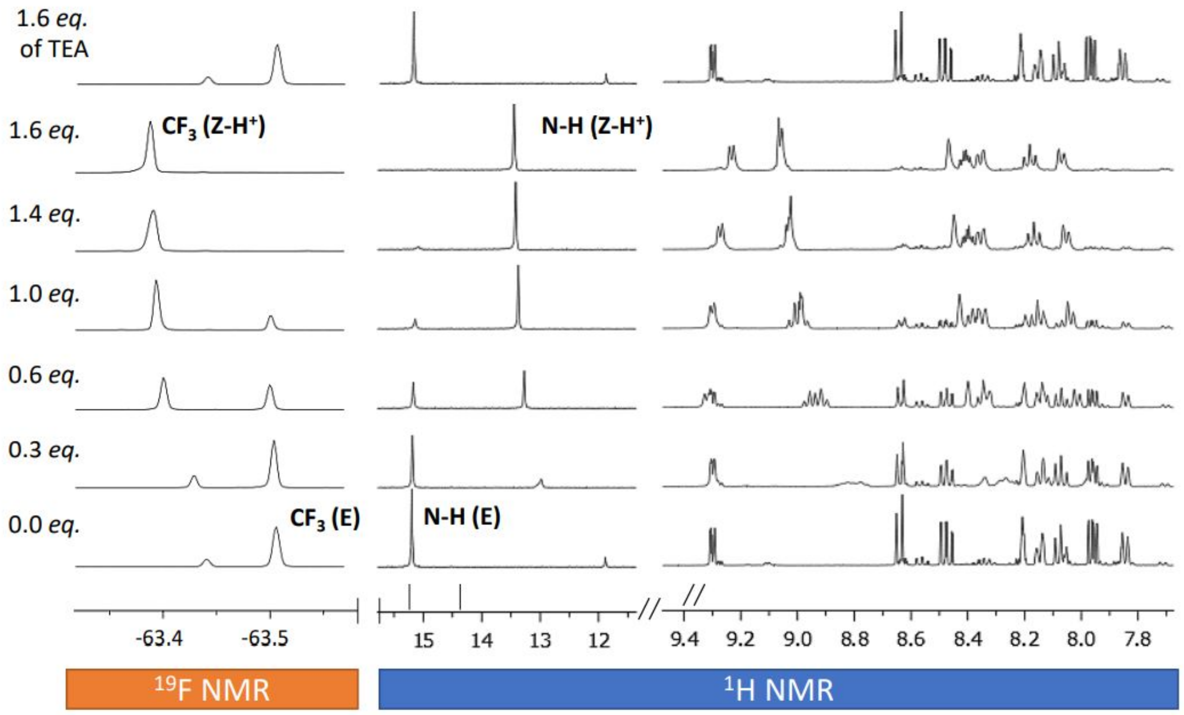

Figure S10. Acid/base switching monitored by ^{19}F and ^{1}H NMR for **2-*E*,** CD_3CN.

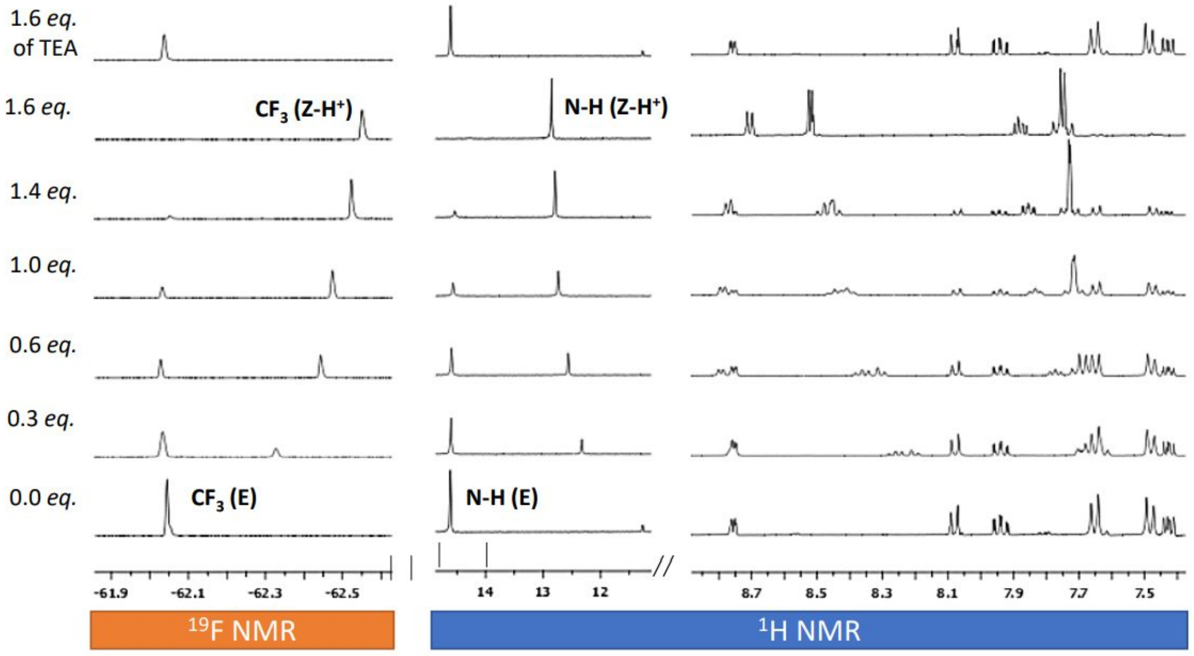

Figure S11. Acid/base switching monitored by ^{19}F and ^{1}H NMR for **3-*E*,** CD_3CN.

4. UV-Vis spectra

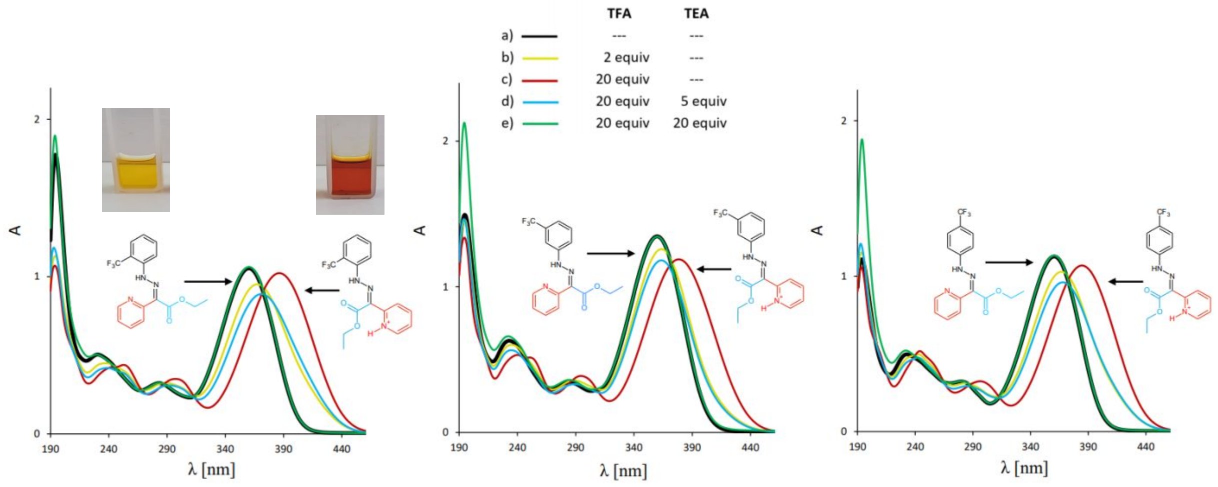

Figure S12. Acid/base switching monitored by UV-Vis for **1-*E***, **2-*E*** and **3-*E*** in CD_3CN.

Table S1. Experimental absorption properties for compounds **1,2** and **3**.

**State**	λ_{max} **[nm]**	ε **[dm^3 mol^{-1} cm^{-1}]**
**1-*E***	361	18305
**1-*Z-H*⁺**	388	16641
**2-*E***	360	23315
**2-*Z-H*⁺**	380	20016
**3-*E***	361	19160
**3-*Z-H*⁺**	385	17821

5. Single Crystal Diffraction

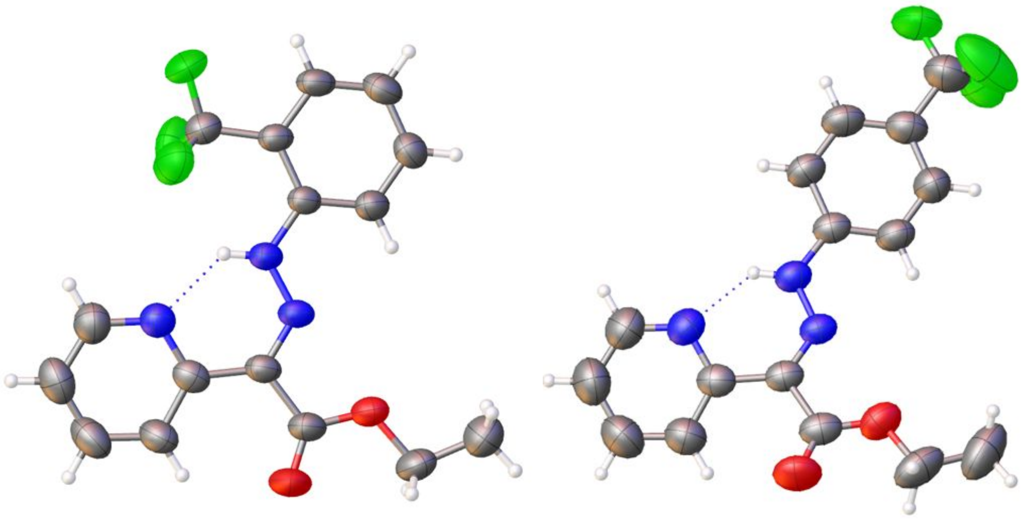

Figure S13. ORTEP drawings (50% probability ellipsoids) of **1-*E*** and **3-*E***. The protons are placed in calculated positions.

X-ray quality crystals were obtained by dissolving 30 mg of the pure compounds in a minimum amount of dichloromethane and allowed to slowly crystallize.

The diffraction data for both compounds were collected using an XtaLAB Synergy-I diffractometer with a HyPix3000 hybrid pixel array detector and microfocused PhotonJet-I X-ray source (Cu Kα). The structures were solved using the SHELXT[1] program and refined by the full-matrix least-squares procedure with Olex2.refine in OLEX2 (version 1.5).[2] Multi-scan absorption corrections were applied using the program CrysAlisPro 1.171.40.82a.[3] Non-routine aspects of refinement: The -CF_3 group in **3** exhibited rotational disorder. This was modelled as disorder over two positions, where the ratio of occupation factors was 0.515(5): 0.485(5). The selected crystallographic data:

1, crystal system: chemical formula: $C_{16}H_{14}F_3N_3O_2$, *M*w = 337.30 g/mol, temperature of experiment = 293(2), monoclinic, space group: *Cc*, *a* = 13.8555(4) Å, *b* = 18.4057(2) Å, *c* = 7.8590(2) Å, *b* = 128.257(4) deg., *V* = 1573.78(9) Å^3, *Z* = 4, D_c / g cm^{-3} = 1.423, μ/ mm^{-1} = 1.034, *F*(000) = 696, Data/restraints/parameters = 2804/2/218, Goodness-of-fit (GOF) on F^2 = 1.004, R_1, wR_2 (I > 2σ(I)) = 0.0415, 0.1096, R_1, wR_2 (all data) = 0.0423, 0.1112, CCDC number = 2089068.

3, crystal system: $C_{16}H_{14}F_3N_3O_2$, *M*w = 337.30 g/mol, temperature of experiment = 293(2), monoclinic, space group: *P2₁/c*, *a* =10.37360(10) Å, *b* = 23.2735(3) Å, *c* = 6.97920(10) Å, *b* = 91.5440(10) deg., *V* = 1684.38(4) Å^3, *Z* = 4, D_c / g cm^{-3} = 1.330, μ/ mm^{-1} = 0.966, *F*(000) = 696, Data/restraints/parameters = 3083/40/247, Goodness-of-fit (GOF) on F^2 = 1.077, R_1, wR_2 (I > 2σ(I)) = 0.0405, 0.1191, R_1, wR_2 (all data) = 0.0453, 0.1236, CCDC number = 2089067.

6. Computational Methods

DFT calculations were carried out using B3LYP hybrid functional combined with a 6-31G (d, p) basis set, CPCM(Acetonitrile).[4] All calculations were performed using *Orca 4.1.1* software.[5] Input files and molecular plots were prepared with *Avogadro* software.[6]

Table S2 Calculated structures and HOMO/LUMO orbitals of isomers.

**1-*E***		
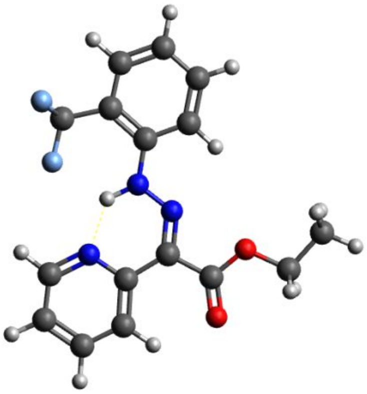	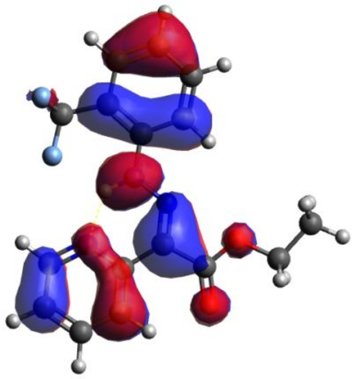	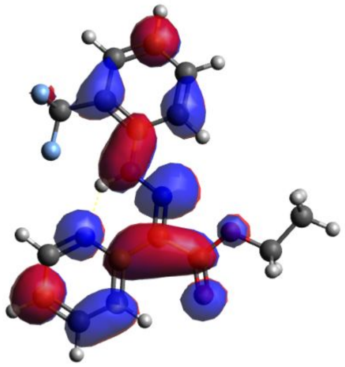

E: 585.634 kJ/mol **HOMO: -5.680 eV** **LUMO: -1.877 eV**

1-*Z*

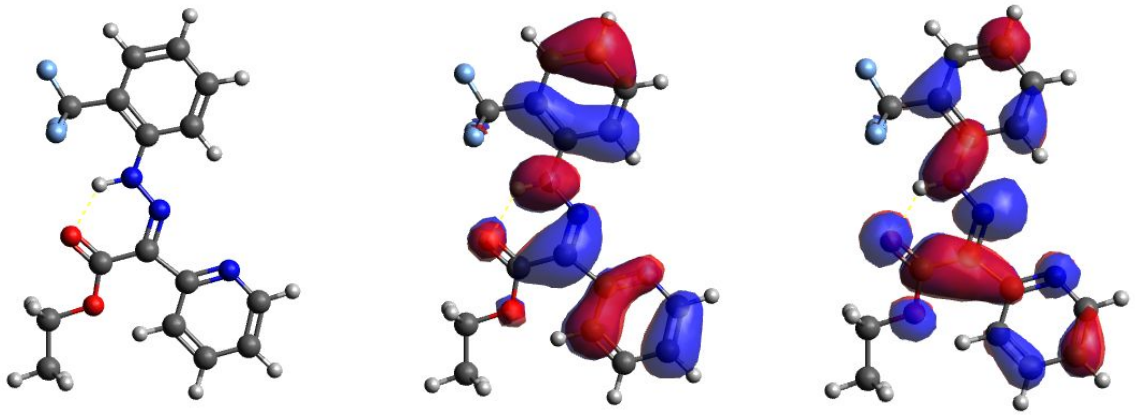

E: 718.924 kJ/mol **HOMO: -5.723 eV** **LUMO: -1.967 eV**

2-*E*

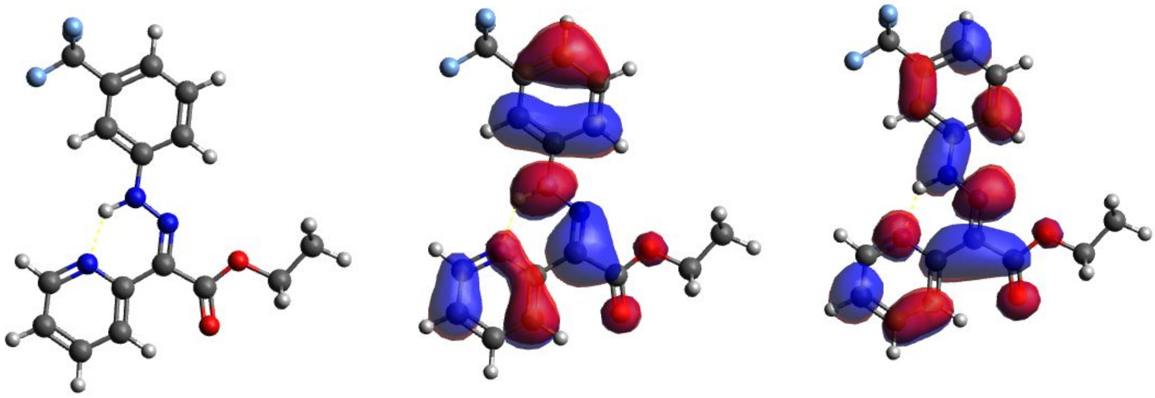

E: 587.68 kJ/mol **HOMO: -5.629 eV** **LUMO: -1.863 eV**

2-*Z*

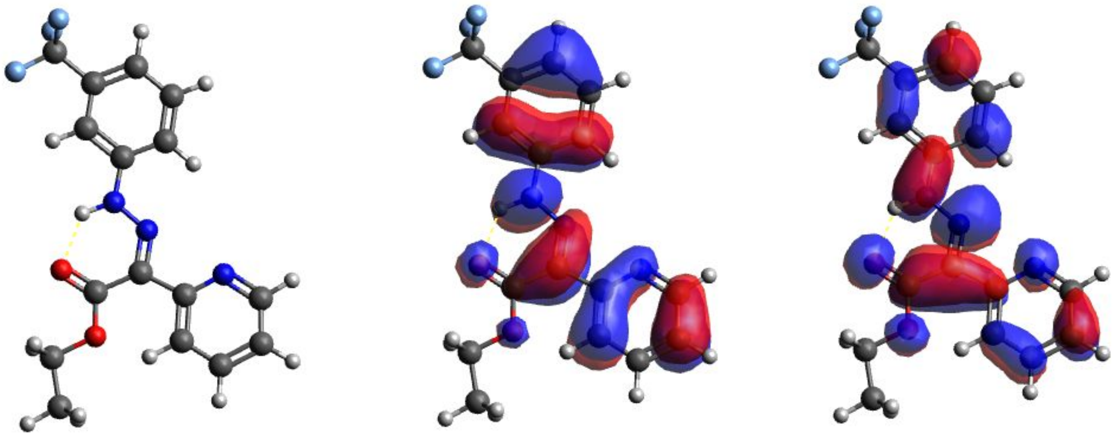

E: 672.156 kJ/mol **HOMO: -5.603 eV** **LUMO: -1.925 eV**

3-*E*

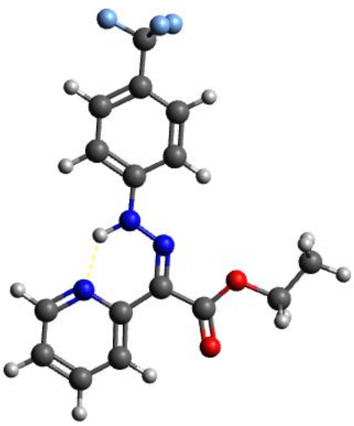

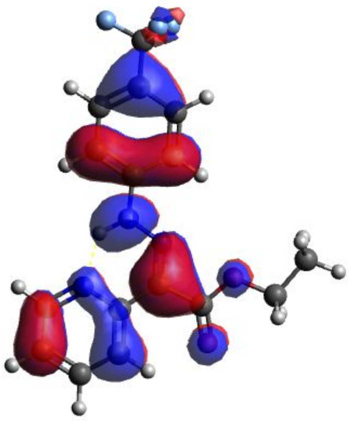

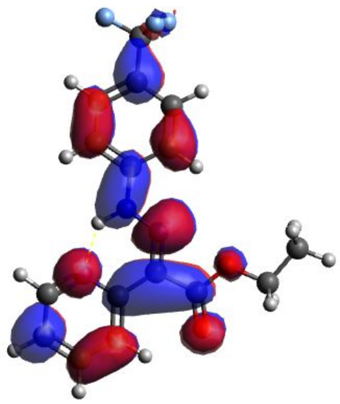

**E: 608.873 kJ/mol**	**HOMO: -5.675 eV**	**LUMO: -1.908 eV**

3-*E*

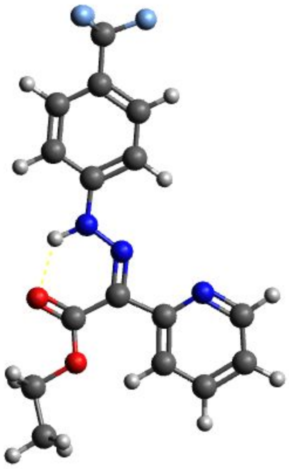

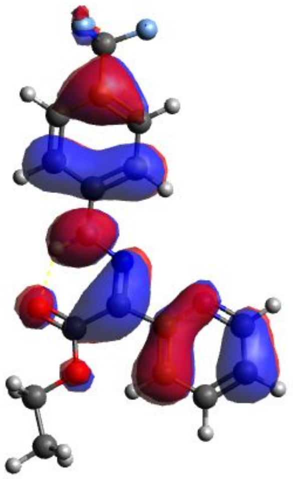

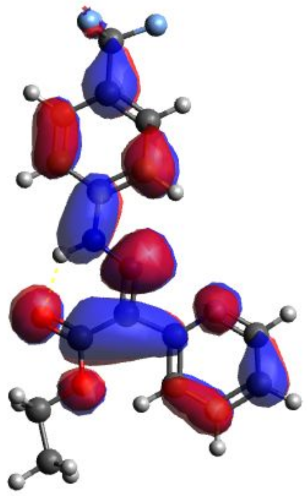

**E: 700.329 kJ/mol**	**HOMO: -5.650 eV**	**LUMO: -1.973 eV**

7. pH-dependent ^{19}F MRI

MRI measurements were performed using a horizontal 9.4T BioSpec 94/20 scanner (Bruker, Germany) running PV5.1 software and equipped with a BFG6-100 gradient coil. To enable rapid high-SNR imaging, a custom-built transmit-receive radiofrequency coil was used. The four-turn strip-solenoid coil with a 12 mm internal diameter was tunable to ^{1}H and ^{19}F nuclei at 400 MHz and 376 MHz, respectively.

The samples for imaging were prepared by dissolving 5 mg of compound **1** or **3** in 1000 μL of acetonitrile. Trifluoroethanol (TFE, 20 μL) was added as a ^{19}F reference. The pH was adjusted to 7, 3.5, and 2 by adding calculated amounts of 0.65 M TFA. First, the scanner was tuned to the TFE resonance frequency. Sample shimming and other adjustments were completed using this frequency. Next, using a single-pulse sequence, the ^{19}F spectrum was acquired, from which relevant frequencies of the –CF_3 group were obtained. For MRI imaging, FLASH (compound **1**) and RARE (compound **3**) sequences were

used. Frequency-selective RF pulses were applied for the selective excitation of individual isomers, thus enabling pH-dependent imaging.

Figure S14 shows the ^{19}F MR images of compound **1** obtained at different pH and different RF transmitter SFO1 frequencies. In the rightmost column, the transmitter frequency shift is provided. Based on the NMR spectrum, the frequency Δ_1 was set to the resonance frequency of the -CF_3 group (blue rectangle, plot S14-C). Image S14-B was taken with this frequency, while for image S14-A, the frequency was changed by 1.8 ppm to cover the spectral region of the second peak (red rectangle). At a neutral pH, there was only one peak, and image S14-A shows only noise.

In the second row, images at the intermediate pH are shown. In this case, the NMR spectrum contained two peaks for the –CF_3 group. For image S14-E, the frequency Δ_1 was set to the resonant frequency of the right peak. Next, the SFO1 frequency was shifted by 1.8 ppm, and image S14-D was acquired.

Finally, for the third row of the table, SFO1 was set to Δ_1+1.8 ppm (image S14-G) and next to Δ_1 for the S14-H image. This is how the images that make up the columns (A, D, G and B, E, H) represent pH-dependent MRI imaging. The FLASH sequence with the following parameters was used to obtain the images shown in Figure S14: TR 33 ms; TE 16 ms; FA 15°, MTX 32 x 32; FOV 3 x 3 cm; NA 900; transmitter excitation pulse bandwidth 200 Hz; receiver effective spectral bandwidth 50k Hz.

The same principles outlined above were used in the MRI imaging of compound **3**, presented in Figure S15. In this case, since the two isomer peaks are closer than in the case of compound **1**, the RF pulses with a narrower bandwidth of 50 Hz and narrower receiver effective spectral bandwidth of 3.2 kHz were used. The RARE sequence with the following parameters was used to obtain the images shown in Figure S15: TR 5000 ms; TE 24 ms; MTX 32 x 32; FOV 3 x 3cm; NA 4; transmitter excitation pulse bandwidth 50 Hz; receiver effective spectral bandwidth 3.2 kHz.

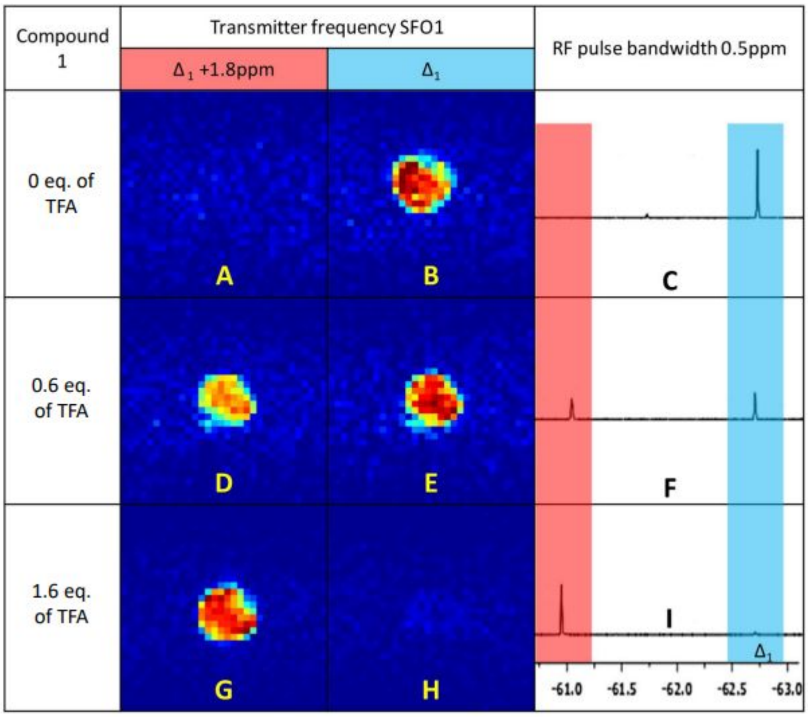

Figure S14. ^{19}F MRI of compound **1**.

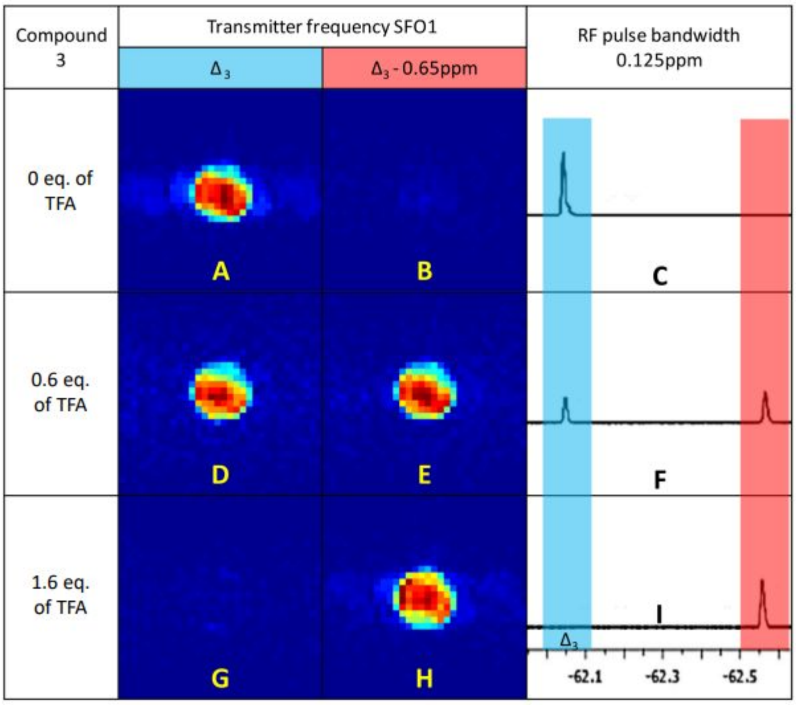

Figure S15. ^{19}F MRI of compound **3**.

8. Relaxation times

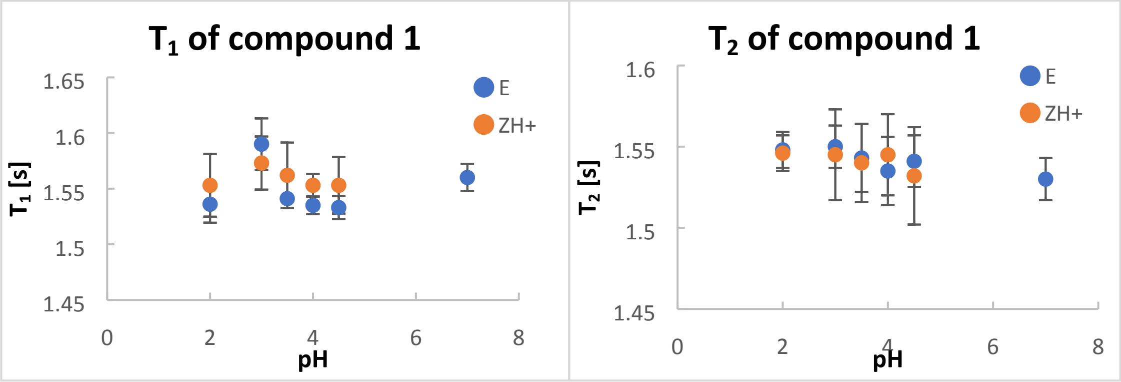

Figure S16. [19]F Relaxation times of compound **1** isomers with pH changes. For each measurement of the relaxation time, 16 experiments with 4 scans were performed.

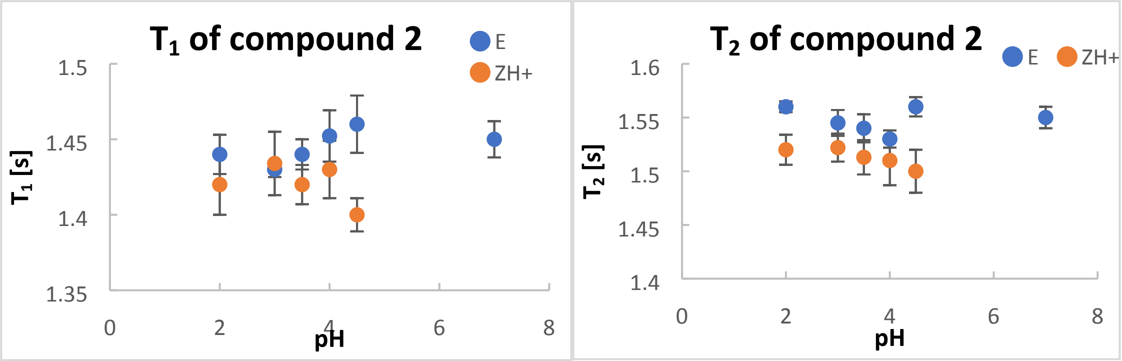

Figure S17. [19]F Relaxation times of compound **2** isomers with pH changes. For each measurement of the relaxation time, 16 experiments with 4 scans were performed.

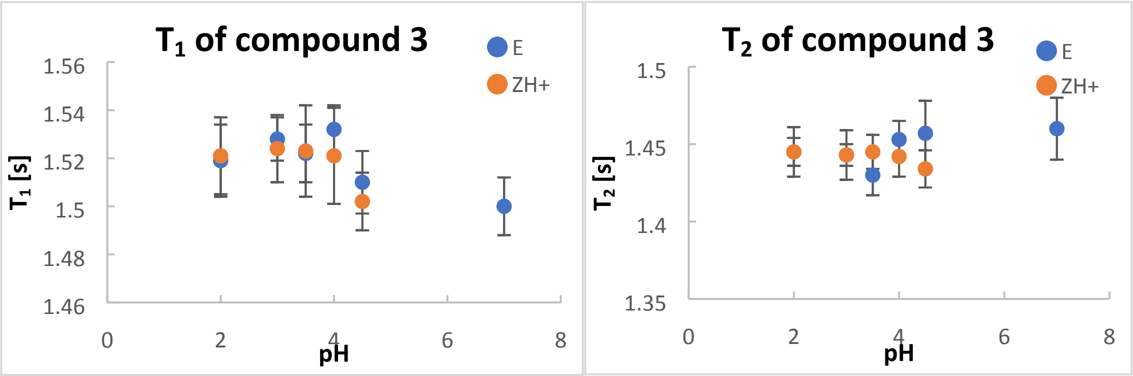

Figure S18. [19]F Relaxation times of compound **3** isomers with pH changes. For each measurement of the relaxation time, 16 experiments with 4 scans were performed.

Table S3. T_1 ^{19}F relaxation times of compounds **1**, **2** and **3**.

Compound	E, pH=7	E, pH=3	ZH+, pH=4.5	ZH+, pH=2
**1**	1.56±0.01 s	1.59±0.02 s	1.55±0.02 s	1.55±0.02 s
**2**	1.45±0.01 s	1.43±0.01 s	1.40±0.01 s	1.42±0.02 s
**3**	1.50±0.01 s	1.53±0.01 s	1.43±0.02 s	1.52±0.02 s

Table S4. T_2 ^{19}F relaxation times of compounds **1**, **2** and **3**.

Compound	E, pH=7	E, pH=3	ZH+, pH=4.5	ZH+, pH=2
**1**	1.53±0.01 s	1.55±0.02 s	1.53±0.02 s	1.54±0.02 s
**2**	1.55±0.01 s	1.54±0.01 s	1.50±0.01 s	1.52±0.02 s
**3**	1.49±0.01 s	1.44±0.01 s	1.43±0.02 s	1.45±0.02 s

9. Solubility

The solubilities of compounds **1**, **2**, and **3** were practically identical and are summarized in Table S5.

Table S5. Solubility of the base (***E***) and protonated (***Z-H⁺***) forms of compounds ***1*–*3*** in various solvents.

**Solvent**	**Configuration *E***	**Configuration *Z-H⁺***
Water	~ 0 mg/ml	~ 3 mg/ml
Ethanol	~ 20 mg/ml	~ 30 mg/ml
Acetonitrile	> 40 mg/ml	> 40 mg/ml
Dichlomethane	> 40 mg/ml	> 40 mg/ml
Diethyl ether	> 40 mg/ml	> 40 mg/ml
Hexane	~ 30 mg/ml	< 0.5 mg/ml
canola oil	> 40 mg/ml	> 40 mg/ml

10. Emulsion

An emulsion of compound **1** was prepared by dissolving 20 mg of **1** in 300 ml of canola oil, followed by sonication with the addition of 300 ml of water and 2 mg of Pluronic 127. The whole process was carried out for 1 h at 30 °C.

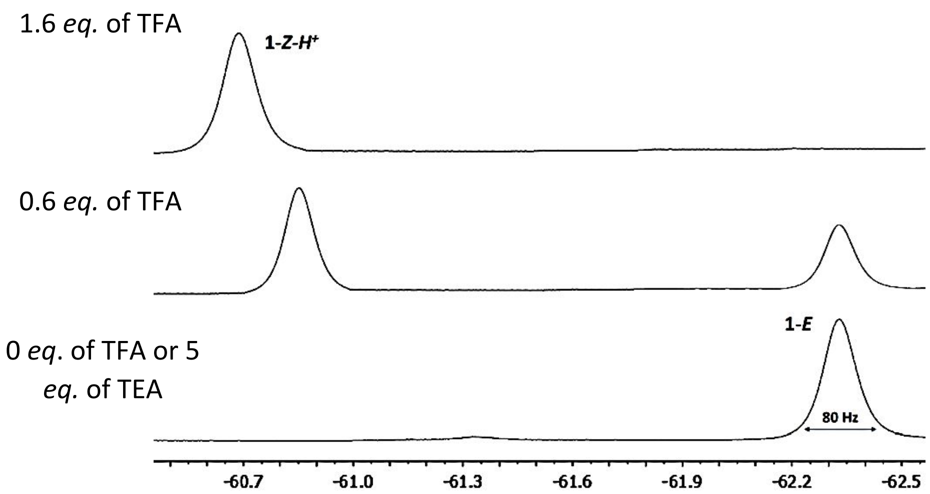

Figure S19. Acid/base switching in emulsion of compound **1** monitored by ^{19}F NMR**.**

The canola oil emulsion containing compound 1 was imaged similar to the previous manner (section 7). Due to the better solubility of the compound in oil, it was possible to use a larger RF coil and to perform simultaneous imaging of samples with different pH. ^{19}F MRI imaging was performed using a double-tuned, ^{1}H/19F, 40mm-diameter, linear, birdcage coil (Bruker T20013V3). The samples were

contained in 25mm long vials cut from NMR tubes containing about 200 µl of emulsion. Figure S20 shows the spatial arrangement of samples with different pH.

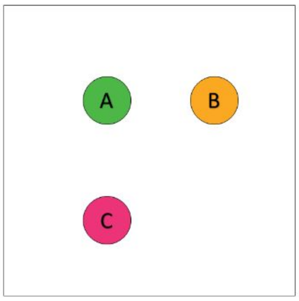

Figure S20. The arrangement of the samples used for ^{19}F MRI. 0 eq. (A, neutral pH), 0.6 eq. (B, 3.5 pH) and 1.6 eq. (C, 2 pH) of TFA.

To obtain the ^{19}F images shown in Figure S21 the RARE sequence with the following parameters was used: TR 5000ms; TE 24ms; MTX 32x32; FOV 3x3cm; rare factor 8; NA 16; transmitter excitation pulse bandwidth 300Hz; receiver effective spectral bandwidth 3.2kHz. To use the pH-dependent resonance frequency shift effect, images were acquired with different RF transmitter SFO1 frequencies. For image S21-A scanner frequency was set to -CF3 group resonance around -62.0 ppm. In this image, a stronger signal from a neutral pH sample and a weaker signal from a 3.5pH sample is observed. To obtain the S21-B image, scanner frequency was moved downfield by 1.7ppm to cover the resonance frequency of the 2pH sample. Indeed in figure S21-B, a stronger signal from a 2 pH sample and a weaker signal from a 3.5pH sample is observed. The images in Figure S21 show how by varying the scanner frequency accordingly, pH-sensitive MRI imaging can be accomplished.

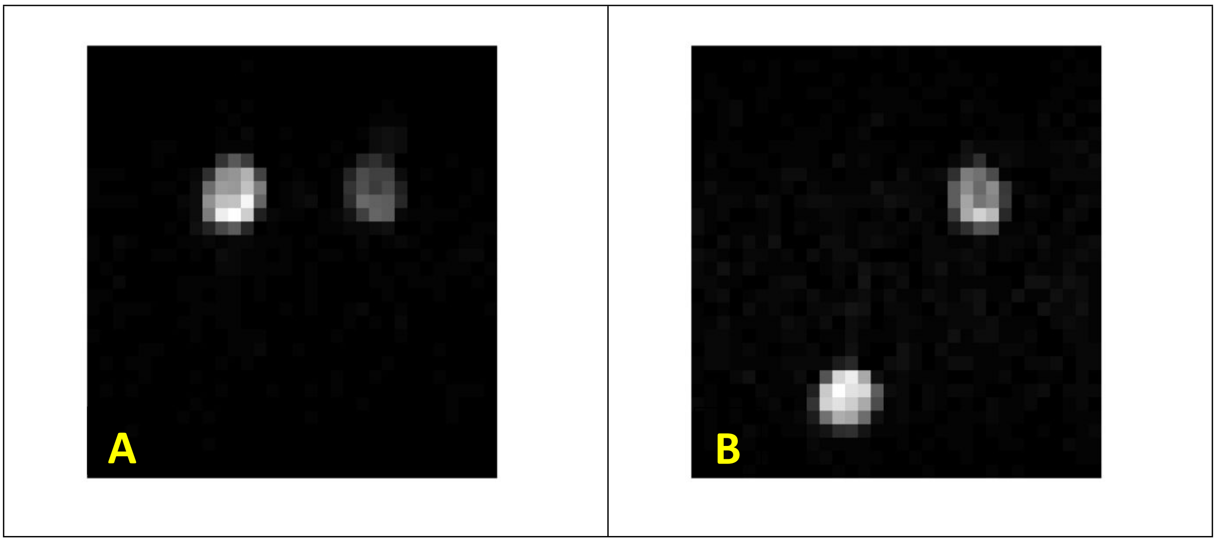

Figure S21. ^{19}F MRI images of three samples obtained with different transmitter frequencies.

References

1. G. M. Sheldrick, *Acta Crystallogr. Sect. A Found. Adv.*, **2015**, *71*, 3.

2. O. V. Dolomanov, L. J. Bourhis, R. J. Gildea, J. A. K. Howard, and H. Puschmann, *J. Appl. Crystallogr.*, **2009**, *42*, 339.

3. Rigaku Oxford Diffraction, **2020**.

4. T. Yanai, D. P. Tew, and N. C. Handy, *Chem. Phys. Lett.*, **2004**, *393*, 51.

5. F. Neese, *WIREs Comput. Mol. Sci.*, **2018**, *8*.

6. M. D. Hanwell, D. E. Curtis, D. C. Lonie, T. Vandermeersch, E. Zurek, and G. R. Hutchison, *J. Cheminform.*, **2012**, *4*, 17.
